# Decoding the reproductive microbiome: enabling clinical and biological insights through machine and deep learning

**DOI:** 10.3389/fendo.2026.1812407

**Published:** 2026-06-15

**Authors:** Ignacio Garach Vélez, Irene Leonés-Baños, Bárbara A. Folch, Laura Antequera, Ignacio Rojas, Francisco Ortuño, María José Sáez Lara, Signe Altmäe, Luis Javier Herrera

**Affiliations:** 1 Department of Computer Engineering, Automatics and Robotics, CITIC, University of Granada, Granada, Spain; 2 Department of Biochemistry and Molecular Biology, Faculty of Sciences, University of Granada, Granada, Spain; 3 Instituto de Investigación Biosanitaria ibs.GRANADA, Granada, Spain; 4 Division of Obstetrics and Gynecology, Department of Clinical Science, Intervention and Technology CLINTEC, Karolinska Institutet, Stockholm, Sweden; 5 Department of Gynecology and Reproductive Medicine, Karolinska University Hospital, Huddinge, Stockholm, Sweden

**Keywords:** biomarker identification, data integration, deep learning, machine learning, microbiome, microbiota, multi-omic analysis

## Abstract

**Background:**

Technological advances have revolutionised the microbiome research in the field of human reproduction, identifying the microbiome as important regulator of reproductive health and functions, where microbes are now linked to sperm quality, ovarian function, endometrial receptivity, embryo implantation, and pregnancy outcomes, including miscarriage and preterm birth. However, the field faces a ‘descriptive phase’ due to the fragmentation of datasets and a lack of functional integration. To progress towards broader understanding and clinical utility, robust computational frameworks are required to translate complex microbial signatures into predictive insights.

**Methods:**

This review classifies the application of machine learning (ML) and deep learning (DL) into essential methodological pillars. We evaluate the entire computational workflow from sequencing data generation to predictive modelling, including data pre-processing for sparsity and compositionality, exploratory, diversity, functional and differential abundance analyses, and advanced data integration to harmonise data across samples, independent datasets, and multiple 16S regions. Particular emphasis is placed on feature selection for biomarker signatures, synthetic data generation, and phenotype classification. Where reproductive-specific evidence is currently limited, normally due to scarce study cohorts, we present proof-of-concept studies from other human niches to demonstrate potential applications. Furthermore, we address the need for Explainable Artificial Intelligence (XAI) to ensure biological interpretability and guide clinical decisions effectively.

**Conclusion:**

Transitioning from descriptive to predictive reproductive medicine relies on integrating ML/DL approaches with biological knowledge. Challenges like small cohort sizes can be overcome through the integration and harmonisation of independent datasets. On a methodological level, researchers should adopt ensemble-based differential abundance and feature selection to reduce data and tool-specific biases. Crucially, any steps altering data nature—such as synthetic data generation—must be strictly confined to training sets to prevent data leakage and preserve model validity. Alongside these technical precautions, standardising analytical workflows and prioritising interpretability are key practical steps. While clinical translation requires extensive validation, moving toward predictive studies is a fundamental first step for future personalised reproductive care. With the current review, we highlight the methodological considerations of ML/DL and provide recommendations for future microbiome studies in the field.

## 1 Introduction

The microbiome plays an important role in reproductive health and disease, embracing hundreds of microbes inhabiting the male and female reproductive tracts (1–3). Despite the importance of these microbes, knowledge of the causes and consequences of dysbiosis, as well as an understanding of their functions and interactions with the host, remains limited. With the increasingly growing number of microbiome studies in the field, a plethora of omics data has been generated; however, reproductive studies are often limited by low biomass, small cohort sizes and a predominantly descriptive approach. To fully leverage the potential of microbiome research in the field, there is a critical need to gather the generated datasets and to apply advanced computational intelligence approaches, such as ML and DL methods, which are becoming standard in biological research (4).

Unlike traditional statistics, ML identifies non-linear patterns and predicts clinical outcomes, such as reproductive success, by automatically adjusting internal parameters based on high-dimensional microbiome profiles. Building upon this concept, DL is a specialised field of ML, inspired by the structure of the human brain. It employs neural networks (NNs), stacked layers of interconnected “neurons” that work together to extract meaningful representations from raw, complex data (5), such as high-dimensional amplicon counts. This learning process enables DL models to capture subtle microbial interactions and host responses, although they are highly data-intensive.

In contrast to the well-studied gut microbiome, the reproductive tract is in some parts a low-biomass environment. This is particularly evident in the female upper tract, as well as in the male reproductive system—including both semen and testicular tissue—making sequence data extremely susceptible to reagent contamination and background noise (1, 3). Further, the extreme taxonomic dominance of *Lactobacillus* in the lower female reproductive tract and the lack of standardised sampling protocols across different anatomical sites pose severe challenges to data processing and require special treatment (2). Algorithms must account for these specific challenges, while also dealing with the common physiological dynamics of women: cyclical hormonal fluctuations, pregnancy phases and age-related shifts in the microbiome depending on whether women are in their reproductive age, peri-menopausal, or post-menopausal. Similarly, male sampling introduces unique variability; for instance, the period of sexual abstinence prior to collection can significantly alter both biomass and microbial composition (6).

**Figure 1:**
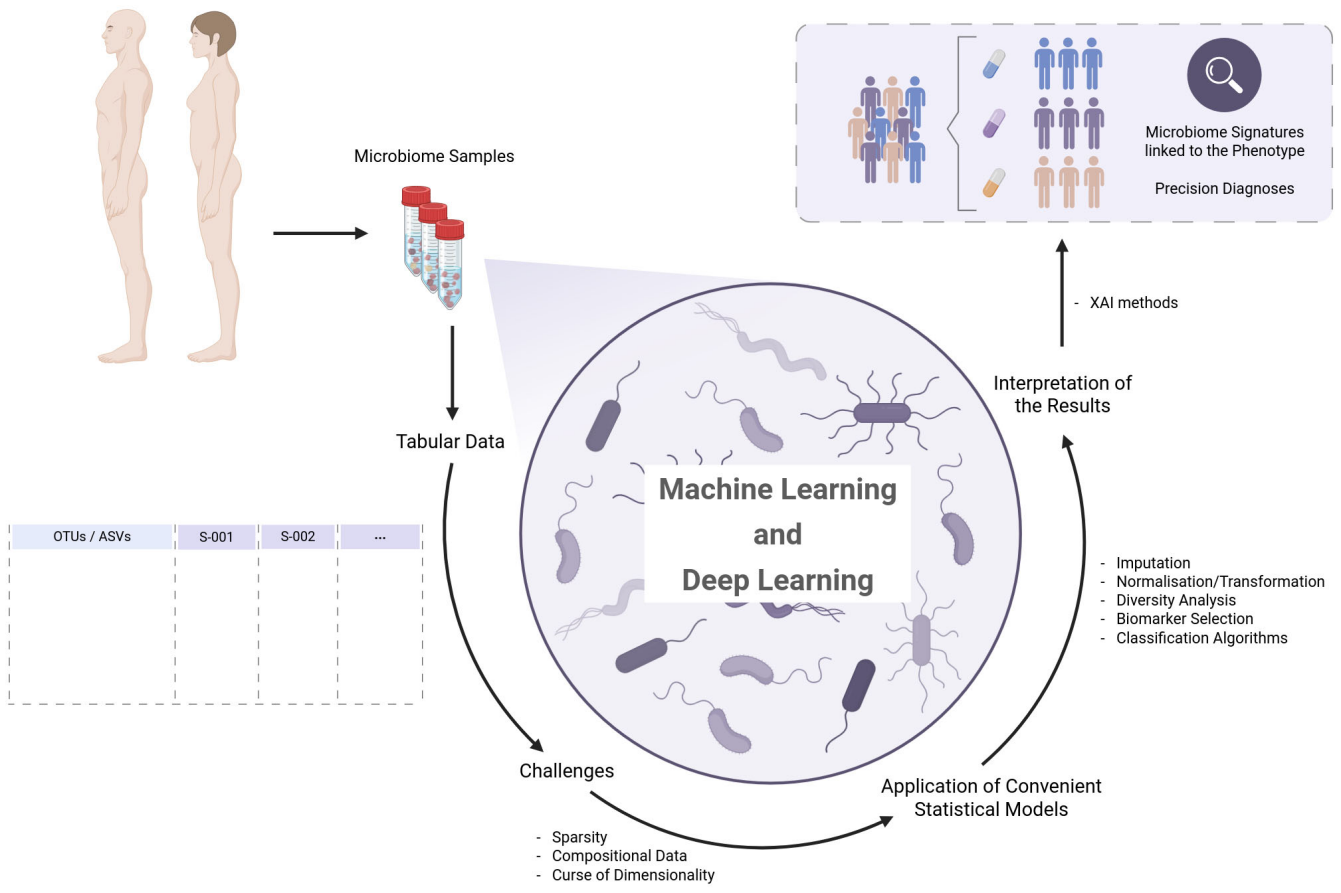
Graphical abstract. The power of machine learning and deep learning in advancing the understanding of the microbiome and its implications for human reproductive health. ASV (Amplicon Sequence Variants); OTU (Operational Taxonomic Units); XAI (Explainable Artificial Intelligence). Created with Biorender.com.

**Figure 2:**
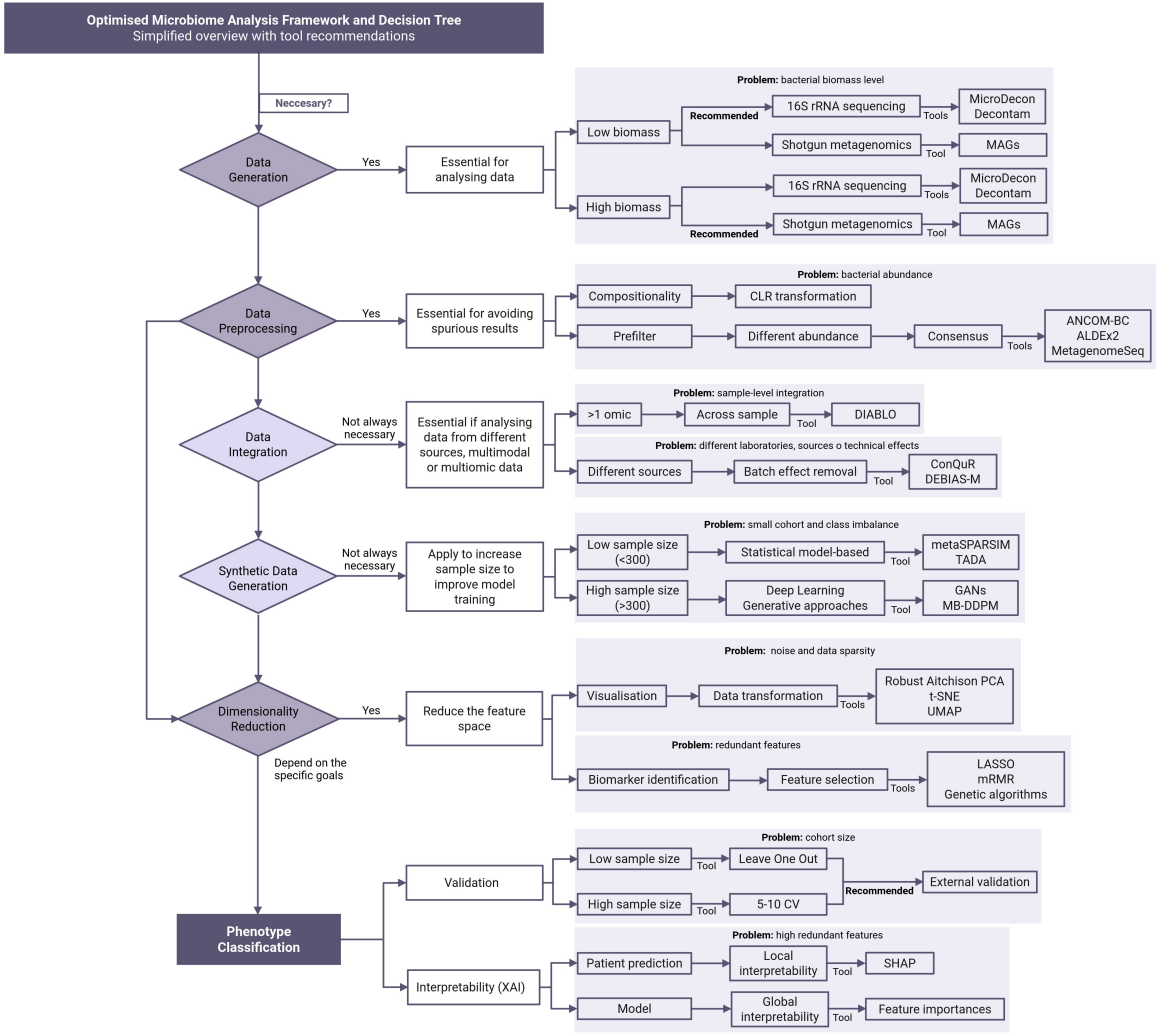
Flowchart providing step-by-step outline of procedures for conducting advanced data analysis on microbiome data, where the problems and recommendations are integrated. Created with Biorender.com.

The methodological landscape for managing these data encompasses several critical stages: pre-processing, multi-omic and multi-dataset integration, synthetic data generation for cohort expansion, and dimensionality reduction for biomarker discovery (**Figures 1**, **2**). Each stage faces domain-specific challenges, such as correcting for kit contaminants in samples with low microbial load, and harmonising heterogeneous datasets across different vaginal or seminal niches. Additionally, the aforementioned hormonal changes and autocorrelations in reproductive data from the same individuals must be carefully accounted for in the predictive layer to avoid introducing bias into the models and their performance evaluation.

With this review, our aim is to highlight the intrinsic characteristics and challenges of reproductive microbiome data, discuss a range of ML and DL approaches, and provide a comprehensive state-of-the-art overview of microbiome research with particular relevance to reproductive medicine. We place special emphasis on Explainable Artificial Intelligence (XAI) to ensure that computational findings remain biologically interpretable and meaningful for clinicians. We further characterise the unique properties of reproductive microbiome data and critically evaluate current analytical methodologies. Importantly, we also identify areas where the application of these advanced techniques within reproductive contexts remains in its infancy. In such instances, we draw upon pioneering studies from other human microbiome niches to illustrate the untapped potential these methods hold for reproductive data. As the field matures, the shift from purely descriptive studies toward predictive and functional analyses is a transition that is not only timely but essential.

By offering this synthesis of current knowledge, our goal is to support researchers in selecting appropriate analytical strategies, enabling them to fully exploit the potential of increasingly complex and valuable microbiome datasets, and to accelerate the discovery of robust, interpretable biomarker signatures that could eventually inform diagnostic development (see **Figure 2** for the workflow and recommendations).

## 2 Sequencing data generation

Generation of sequencing data allows to work with a tabular database representing each of the sequenced microorganisms (clustered as Operational Taxonomic Units [OTUs] or resolved into Amplicon Sequence Variants [ASVs]) and samples according to their abundance, organised in rows and columns. Detailed information of the microbiome data generation is summarised in previous works (3, 7). 16S rRNA gene hypervariable region sequencing studies are prevalent in the reproductive field due to the affordable price, sensitivity and robustness of the method. Further, the upper reproductive tract and semen are low microbial biomass sites, where 16S rRNA gene sequencing protocols are well developed, whereas the whole metagenome (i.e., shotgun) sequencing is less sensitive (1, 3, 8, 9). The restraint of 16S rRNA gene analysis, however, is the limited sensitivity in detecting bacterial species, providing a broad microbiome overview but lacking the specificity required for many biological and clinical applications (10). Higher resolution at the species/strain level is essential to fully understand microbiome behaviour, interactions, and impacts on health and disease. The whole 16S rRNA gene amplicon (V1-9) amplification with deep sequencing is a new alternative to identify microbes at the lowest taxonomic levels (11–13). Moreover, in the low microbial biomass sites, combating contamination is crucial in order to detect true microbial reads beyond contamination. Several decontamination tools are available: (i) Decontam (14), which uses a statistical framework to identify and remove contaminant sequences based on their prevalence in negative controls and their inverse correlation with sample DNA concentration; (ii) microDecon (15), which mitigates contamination by modelling the contribution of contaminant taxa from blank samples to subtract them from true sample profiles; (iii) MicrobIEM a recently developed tool (16) that offers an intuitive graphical interface for users without coding experience, and introduces novel filtering approaches based on the ratio of relative abundances and the consistency of contaminants across negative controls, providing interactive plots and flexible parameter settings to support quality control; (iv) HoCoRT tool (17) simplifies the process of removing host contamination from sequencing data by integrating multiple well-established tools into a single pipeline to classify sequences into host and non-host origin.

For the abundant microbial biomass sites, such as vagina-cervix, shotgun metagenomic sequencing offers taxonomic resolution down to the strain level that enables identification of functional and metabolic pathways involved. Furthermore, shotgun sequencing allows the profiling of non-bacterial organisms, including viruses, fungi, archaea and protozoa. Nevertheless, shotgun metagenomics sequences all available DNA, meaning samples are often contaminated with host reads. Therefore, applying host depletion tools such as HoCoRT (17), is essential to isolate the true microbial signal prior to analysis. Recent breakthroughs in high-throughput sequencing technologies and metagenomic binning techniques enable the large-scale assembly of nearly complete Metagenome-Assembled Genomes (MAGs), where each MAG constitutes a comprehensive genomic representation of individual organisms (18). Metagenomic binning techniques facilitate the discovery of numerous previously unidentified bacterial species within the microbiome and allow the detailed characterisation of microorganisms’ evolutionary and functional differences at the genomic level (19). MAGs represent a paradigm shift, providing a more comprehensive and precise characterisation of the human microbiome, allowing the direct study of genomes from uncultured microorganisms present in biological samples (20). In the reproductive field, MAGs have enabled strain-level characterisation of the vaginal microbiota in pregnant women, distinguishing commensal from virulent variants. These vagina-specific genomes have revealed key virulence factors and pathogenic potential, improving diagnostic accuracy and the prediction of pregnancy-related complications (21). Furthermore, Jung et al. (22) utilised MAGs to identify microbial signatures associated with high-risk human papillomavirus (HPV) oncogenicity. Their analysis of 137 MAGs revealed distinct microbial profiles across cervical lesion stages, clarifying the microbiome’s role in HPV-related pathogenesis and disease progression. Altogether, MAGs offer transformative potential by providing high-resolution data that clarify pathogen characteristics and reveal microbial signatures associated with disease progression.

## 3 Data preprocessing and data exploration

Effective preprocessing of microbiome data is essential for extracting meaningful biological insights, especially when employing advanced analytical methods such as ML/DL algorithms (23, 24). A key challenge stems from the compositional nature of microbiome data. Traditional statistical models often assume that data are measured on a Euclidean scale, where distances reflect absolute differences. However, microbiome data provide relative abundances—proportions of each taxon relative to the others—without information on absolute quantities, as the values are constrained to sum to one (25). This requires the use of appropriate transformations or normalisation techniques to account for the constant sum constraint and render the data suitable for conventional multivariate analysis (26). The most widely used approaches rely on logarithmic transformations, such as the centered log-ratio (CLR) and the isometric log-ratio (ILR) (27), which map the relative abundances into hyperplane points in the Euclidean space, enabling the application of unconstrained statistical tools.

In some applications—particularly those aimed at identifying rare taxa or linking community structure to phenotypes—the microbiome table is binarised to reflect only presence or absence of taxa. This simplifies the dataset, eliminates compositional constraints, and mitigates biases from sequencing depth variation, enabling predictive performance comparable to that of standard abundance-based transformations (28–30). However, this comes at the cost of losing abundance information, a trade-off that may only be justified during later stages of the analysis. Moreover, different normalisation techniques may be more suitable for each specific algorithm used at biomarker identification and phenotype classification steps (28).

Sparsity is another challenge, characterised by abundant zeros in taxonomic tables. This sparsity in the data reflects the clinical reality of endometrial samples, where the biological signal is so faint that the algorithms must learn to distinguish the actual microbial presence from background noise. Consequently, this distorts distributions and precludes log transformations, which are undefined at zero. A common workaround is the addition of a small pseudocount to enable log transformations, but consensus on the best strategy is lacking (23, 31). Alternatively, tools such as ANCOM-BC (32) implement strategies to distinguish between the structural zeros, sampling zeros, and outliers (33).

Additionally, the compositional structure introduces spurious negative correlations, where an increase in one taxon’s proportion implies a reduction in others (23), even though the absolute abundance has not changed. Ignoring this fact may lead to erroneous clinical interpretations regarding niche displacement. This is particularly relevant given the typical *Lactobacillus* dominance in the lower female reproductive tract. As a result, standard correlation coefficients such as Pearson and Spearman are inappropriate. To address this, the Variance of the Log-Ratio (VLR) has been proposed as a measure of association that avoids the need for prior normalisation (34, 35). However, its interpretation is limited by the lack of a meaningful scale. The propr R package (36) provides a solution by scaling the VLR metric for interpretability. While no novel correlation measures have emerged recently, current efforts focus on leveraging existing methods to construct microbial association networks, which provide insight into the inter-taxa interactions. Tools like NetCoMi (37) allow for the construction, visualisation, and comparison of such networks. These methods help to identify microbial interactions associated with health or disease states, monitor community shifts across hormonal cycles and infer functional dependencies.

### 3.1 Diversity and functional analyses

During sequencing, the number of reads generated per sample can vary considerably, whether using 16S rRNA amplicon sequencing or metagenomic shotgun approaches (26). To ensure meaningful comparisons of microbial diversity across samples, it is essential to minimise biases introduced by differences in sequencing depth. Rarefaction analysis is a widely adopted method for addressing this issue: it standardises sequencing depth by subsampling each sample to a fixed read count threshold and discarding those that fall below it (38). This approach enhances the comparability of bacterial communities and supports reliable downstream diversity and exploratory analyses. However, rarefaction has not been shown to improve phenotype classification performance (28, 30).

Alpha and beta-diversity analyses are standard data exploration tools that provide insights into microbial community structure. Alpha-diversity captures within-sample diversity, incorporating both richness (the number of taxa) and evenness (the relative distribution of taxa). Richness can be measured using indices such as Observed OTUs/ASVs or Faith’s Phylogenetic Diversity, while evenness is represented by Pielou’s and Simpson’s Evenness. Composite metrics like the Shannon and Gini-Simpson indices incorporate both aspects. It is generally recommended to use multiple metrics to capture the multidimensional nature of microbial diversity. Platforms such as QIIME2, via the scikit-bio python library (39), offer tools to compute these indices. The vegan package also stands as an alternative in R (40).

Beta-diversity, in contrast, assesses inter-sample differences in community composition. It quantifies how similar or dissimilar microbial profiles are across samples. Common metrics include the Jaccard index (based on presence/absence), Bray-Curtis dissimilarity (accounts for abundance), and UniFrac (incorporates phylogenetic relationships). These metrics are typically visualised via ordination methods such as Principal Coordinates Analysis (PCoA), enabling researchers to explore microbial shifts between sample groups and their potential implications for reproductive health.

Lastly, functional inference methods provide predictive insight into the metabolic potential of microbial communities. PICRUSt2 predicts functional profiles from 16S rRNA data using ancestral state reconstruction (41). By leveraging phylogenetic placement, it infers the gene content of taxa based on reference databases such as the Kyoto Encyclopedia of Genes and Genomes (KEGG) and Enzyme Commission. Although limited by database completeness, PICRUSt2 provides a useful approximation of community-level functionality.

Alternatively, functional profiling can be achieved with greater resolution using shotgun metagenomics, which enables direct and accurate quantification of functional genes and pathways. One widely adopted pipeline for functional profiling of shotgun metagenomes is HUMAnN (42). HUMAnN quantifies gene families, pathways and Enzyme Commission numbers, by first performing a nucleotide-level search against a pangenome database, followed by unmapped read translation and protein alignment, finally estimating pathway abundance using structured databases. Following this quantification, the functional profiles allow researchers to extract pathways enriched or depleted in different conditions. Similarly, the FMAP tool provides a framework comparable to HUMAnN but includes downstream statistical analyses for further interpretation and group comparisons (43).

### 3.2 Differential abundance

Identifying taxa that differ significantly between conditions of interest is a common step in metagenomic pipelines. However, the previously discussed properties of microbiome data pose considerable challenges. The microbiome data, particularly those representing the abundances of OTU/ASVs, seldom follow a normal distribution due to their complex and diverse nature (25). Therefore, when comparing the abundances of taxonomic units between groups of samples, it is better to use a non-parametric test, such as the Mann-Whitney U test or Kruskal-Wallis test, rather than relying on a test that assumes normality in the data. Nonetheless, when examining relative abundance data, these tests overlook the compositional nature of relative abundances. Additionally, they perform poorly in terms of power and False Discovery Rate (FDR) when sample sizes are small, or data is sparse (23).

To address these issues, a wide array of differential abundance methods have been developed, each incorporating different assumptions and modelling strategies. For example, ALDEx2 (44) and ANCOM-BC (32) explicitly deal with compositionality using log-ratio transformations, while metagenomeSeq (45) uses a zero-inflated gaussian distribution to model the count data, providing robustness against sparsity and variability in sequencing depth.

A recent large-scale study systematically compared the performance of 14 differential abundance tools across 38 16S rRNA datasets, revealing substantial discrepancies in the sets of taxa identified by each tool (46). This heterogeneity can lead to inconsistent and potentially misleading biological interpretations. Their results revealed two major categories of tools: those that identify many features (e.g., limma voom, edgeR, LEfSe, Wilcoxon-CLR) and those that are more conservative (e.g., ALDEx2, ANCOM-BC), the latter tending to detect fewer but more consistently replicated taxa. The remaining methods—DESeq2, MaAsLin2, metagenomeSeq, corncob, and the t-test—fell in an intermediate range. MetagenomeSeq demonstrated controlled FDR in this analysis, though at the expense of low power. The study showed that methods perform variably depending on data filtering, with sparsity and sequencing depth imbalances playing a critical role in false positive rates (46).

A general recommendation would be to adopt a consensus-based or ensemble approach, applying multiple differential abundance methods and selecting taxa based on what the majority agree on (46, 47). This strategy helps mitigate method-specific biases and increases confidence in the selected features. Once the taxa are identified, the data can be readily used for downstream machine learning tasks. However, if the number of selected species is still large, an additional feature selection step is advised before classification, to reduce noise and improve interpretability.

Importantly, when used as a pre-filtering step prior to supervised classification, differential abundance testing must be restricted to the training data in each validation split to avoid data leakage and overoptimistic performance estimates. This consideration represents a key methodological shift when transitioning from descriptive to predictive modelling.

## 4 Data integration

**Figure 3:**
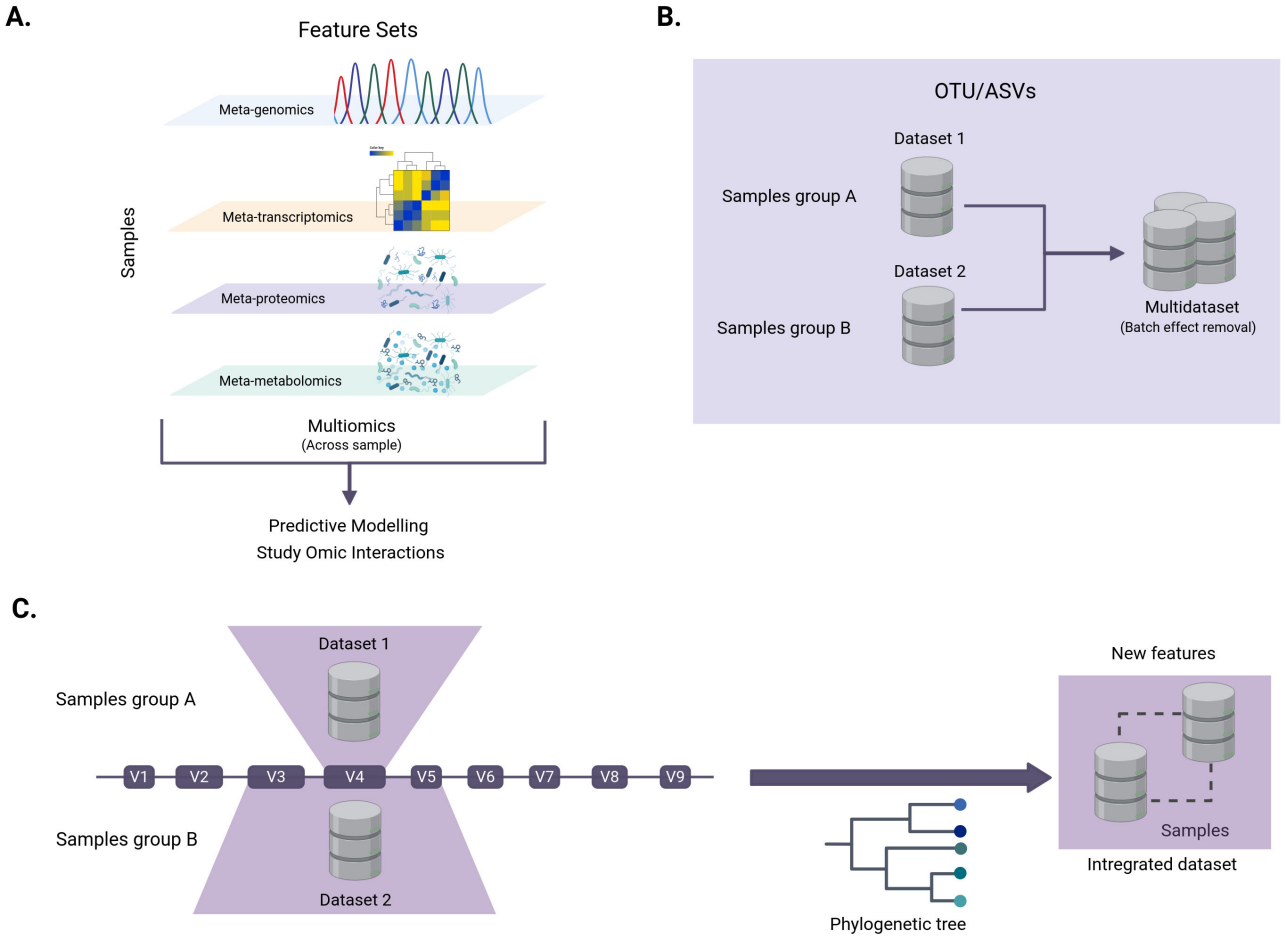
Data integration procedures. **(A)** Multi-omic integration requires various omics analyses for the same individual, allows studying relationships between data types, and provides more powerful predictive modelling using the integrated information. **(B)** Multidataset integration removes technical bias and batch effects, making datasets tractable together for any joint analysis. **(C)** 16S rRNA hyperregion integration is a subtype of B, but in this case not only technical effects, but the different biological sequencing target data is harmonised making datasets comparable. Created with Biorender.com.

To provide a broader understanding of complex biological systems, integrating diverse types of data has become a key strategy in microbiome and biomedical research. In particular, researchers often aim to integrate data across multiple omics layers within the same samples, or across distinct datasets (**Figure 3**). This integrative approach helps to address the common limitation of small sample size in individual studies, which is a critical issue for ML/DL methods.

### 4.1 Across-sample integration

Across-sample or multi-omic integration is particularly relevant in reproductive microbiome research. Microbiome samples from different body sites and various omics analyses can often be collected from the same individual, making it essential to analyse them jointly to better understand local and systemic microbiome interactions. To achieve reliable results, it is crucial to use correctly matched samples and address missing data or modalities before integration, as well as to ensure proper normalisation and scaling across the distinct omics layers. Due to the current scarcity of multi-omic integration studies focused on the reproductive microbiome, some of the methodologies discussed below are further illustrated with examples from other biological niches. However, these applications serve as robust proof-of-concept approaches with clear potential for translation into reproductive contexts. Five recent methods stand out for integrating multi-block datasets collected from the same set of samples (i.e., across-sample integration): Multi-Omics Factor Analysis (MOFA), Hypergraph induced Orthogonal Nonnegative Matrix Factorisation model (HONMF), Joint Negative Binomial Model (JNBM), Data Integration Analysis for Biomarker discovery using Latent variable approaches for Omics studies (DIABLO) and microbe–metabolite vectors (mmvec) (**Table 1**).

**Table 1:** A collection of tools for integrating datasets across omics and studies.

Category	Tool	Method	Modality	Availability
Across-Sample Integration	MOFA [Argelaguet et al., (48)]	Unsupervised (Variational Bayesian inference)	Multi-omics data	Python, R
	HONMF [Ma et al., (49)]	Unsupervised (Hypergraph-induced NMF)	Multi-omics data	MATLAB
	DIABLO [Singh et al., (50)]	Supervised (Latent variable approach for biomarker discovery)	Multi-omics data	R (mixOmics)
	Mmvec [Morton et al., (51)]	Supervised (Neural network for co-occurrence probabilities)	Microbiome and metabolome data	Python
	JNBM [Kim et al., (52)]	Unsupervised (Mixtures of latent factors)	Microbiome and metagenomic data	R
Across-Dataset Integration	ConQuR [Ling et al., (53)]	Supervised (Conditional Quantile Regression)	16S rRNA and metagenomic data	R
	MMUPHin [Ma et al., (54)]	Supervised (Empirical Bayes for batch correction)	16S rRNA and metagenomic data	R
	DEBIAS-M [Austin et al., (55)]	Supervised (Phenotype-aware batch correction)	16S rRNA and metagenomic data	Python
	PLSDA-batch [Wang and Lê Cao, (56)]	Supervised (Multivariate non-parametric)	Omic Datasets	R
Across 16S Region Integration	MaLiAmPi [Minot et al., (57)]	Phylogenetic placement (ASV matching and tree building)	16S rRNA datasets from different regions	nextflow
	SEPP [Janssen et al., (58)]	Phylogenetic placement (Fragment insertion into reference phylogeny)	16S rRNA datasets from different regions	qiime2 plugin

In supervised learning, the algorithm is trained on labelled data, meaning it is provided with input-output pairs to learn the relationship between the input variables and the target variable. In contrast unsupervised learning involves training the algorithm on unlabelled data to learn patterns and structures.

MOFA, Multi-Omics Factor Analysis; HONMF, Hypergraph induced Orthogonal Nonnegative Matrix Factorization model; DIABLO, Data Integration Analysis for Biomarker discovery using Latent Components; mmvec, Microbe–Metabolite Vectors; JNBM, Joint Negative Binomial Model; ConQuR, Conditional Quantile Regression; MMUPHin, Meta-analysis Methods with Uniform Pipeline for Heterogeneity in Microbiome Studies; DEBIAS-M, Domain adaptation with phenotype Estimation and Batch Integration Across Studies of the Microbiome; PLSDA-batch, Partial Least Squares Discriminant Analysis with batch correction; MaLiAmPi, Maximum Likelihood Amplicon Pipeline; SEPP, SATé-Enabled Phylogenetic Placement.

MOFA is implemented using variational approximate Bayesian inference, to extract latent factors shared across omic layers (48). In the gut, MOFA has been employed for the integrative analysis of multimodal microbiome data in critically ill sepsis patients and healthy volunteers, revealing cross-kingdom microbiome disruptions following broad-spectrum antibiotic exposure (59). This approach serves as inspiration for investigating similar microbial network disruptions in the vaginal or endometrial microbiome following antibiotic treatments for urogenital infections. In contrast, HONMF allows the matrix of low-dimensional representation (latent variables) to vary for each composition profile dataset, which might be more appropriate to integrate cross-modality information due to batch effects (49). A sample clustering analysis based on the learned low-dimensional factor matrix on three datasets (gut, sputum and soil) was conducted, HONMF steadily separated the groups, outperforming MOFA and other unsupervised methods in concordance scores with the ground-truth groups (49). However, HONMF is implemented in MATLAB, which may pose accessibility limitations relative to other methods in this section that are available in commonly used languages such as R and Python.

Taking a different statistical approach, the JNBM provides another robust framework for paired microbiome sequencing data collected across various body sites (52). By modelling using mixtures of latent factors, it allows considering heterogeneity among the subjects in cross-site associations. A major benefit of JNBM is its ability to improve predictive performance, enabling researchers to predict microbial abundances at one site based on observations from another. The utility of this model was demonstrated in a case study analysing paired vaginal and urine samples. JNBM revealed distinct positive associations of age and body mass index with *Streptococcus* and *Gardnerella* abundances across both niches, associations that were not revealed when performing an isolated analysis (52).

On the other hand, DIABLO is a method that identifies biologically relevant features and at the same time it performs supervised learning allowing to make predictions on new samples (50, 60). In its operation, the DIABLO framework assumes a linear relationship between the selected features and the resulting phenotype. DIABLO was used in an extensive analysis (61) that integrated bacterial/viral metagenomic data and different HPV genomes, revealing bacterial taxa that were associated with bacterial vaginosis (BV) and also correlated with the presence of HPV.

To address the limitations of correlation-based approaches—mainly the already mentioned compositional issue— in multi-omics integration, mmvec, a neural network approach implemented in Python and also available as a plugin within QIIME2, was introduced to model the co-occurrence probabilities between microbial and metabolite features (51). This approach sidesteps issues related to differing measurement scales between the sequencing and mass spectrometry. By learning conditional probabilities (e.g., the likelihood of observing a metabolite given a microbe), mmvec provides interpretable insights into the microbe–metabolite interactions, while its core probabilistic framework is conceptually adaptable to other omics—for instance, to model the association between microbial taxa and protein expression patterns. A previous study successfully identified interactions between the metabolites and microbial taxa in the cervicovaginal microenvironment that were predictive of genital inflammation and disease status (62).

In addition to these tools, there are emerging ML/DL strategies and techniques, not implemented in specific software packages, which facilitate data integration for microbiome research (63, 64). For instance, a previous study employed a deep neural network-based variational autoencoder (VAE) to extract latent features from multi-omic high-dimensional data (RNAseq, miRNA, methylation and Copy Number Variation). The subsequent integrated multi-omics analysis of ovarian cancer obtained near perfect cancer samples classification performance (64). These DL approaches are usually omic-agnostic, meaning they can accept any set of microbial features alongside other omics data as input. The autoencoder generates vectors that capture an integrated representation of all inputs; these representations tend to be significantly more robust when dealing with missing data (a common situation in multi-omic studies), making these models suitable for joint analysis using either supervised or unsupervised techniques. Ultimately, these techniques are highly adaptable to complex reproductive conditions like endometriosis. To illustrate, an autoencoder could integrate endometrial microbiome profiles with host transcriptomics and local metabolomic data, uncovering hidden interactions that may drive inflammation, even when some of the patient data modalities are incomplete.

### 4.2 Across-dataset integration

An essential consideration when integrating microbiome datasets is the presence of batch effects, which can arise from diverse sources, including different sequencing platforms, DNA extraction protocols, primer sets, use of negative/positive controls, or bioinformatics pipelines, each introducing its own layer of technical variation. Effective removal or correction of such effects is crucial to ensure validity and comparability of downstream analyses, particularly in large-scale meta-analyses or cross-cohort studies.

Several techniques have been proposed to reduce this unwanted variability, supporting the accuracy of the attained results and reinforcing the reliability of the findings (**Table 1**).

ConQuR (Conditional Quantile Regression), a two-part non-parametric approach is specifically designed to correct microbiome batch effects (53). ConQuR models the zero-inflated distribution of microbial read counts by combining logistic regression for presence-absence modelling and quantile regression for non-zero counts. This method was applied at a large-scale meta-analysis of over 10,000 microbiome samples from pregnant women across the gut, vagina, and oral cavity (65), revealing specific gut trajectories, dynamic vaginal shifts and different microbial signatures linked to gestational diabetes and preterm birth, thus offering a comprehensive atlas of the pregnancy microbiome.

For large meta-analyses, MMUPHin (54) offers a single statistical system, including batch correction, differential abundance testing and population structure identification. Its batch correction module accounts for zero inflation and estimates batch-specific parameters using empirical Bayes. This model cautiously adjusts for technical variability while preserving meaningful biological signals and accounting for confounders, as was demonstrated in a meta-analysis of the gut samples from Inflammatory Bowel Disease (IBD) patients (54), highlighting the tool’s potential for performing similar meta-analyses using vaginal or endometrial samples from different cohorts. Using these integrative approaches enables the augmentation of the typically small sample sizes on endometriosis cohorts, providing a path to uncover new insights of the disease.

Another tool for across-dataset integration is DEBIAS-M (55). DEBIAS-M learns interpretable microbe and batch-specific correction factors that reduce batch effects while preserving cross-study phenotype associations. Compared to ConQuR and other integration methods, DEBIAS-M improved cross-study cervical neoplasia classification based on 323 cervicovaginal microbiome samples from five technically diverse studies (55).

Lastly, PLSDA-batch adapts Partial Least Squares Discriminant Analysis for batch correction (56). It sequentially estimates and subtracts batch-associated components after controlling for treatment variation, thus preserving the biologically relevant signals. Variants of PLSDA-batch are available to handle unbalanced designs and reduce overfitting through variable selection, making it particularly suitable for experimental setups with overlapping treatment and batch effects. Although not yet applied to reproductive data, its potential is highlighted by a recent head and neck cancer study (66), in which PLSDA-batch was used to integrate 16S rRNA oral microbiome datasets from 12 studies involving 938 samples. This tool was able to identify a shared microbial profile—something previous studies had failed to achieve due to inconsistent datasets.

### 4.3 Across-16S region integration

Recent efforts to enable cross-study integration of the abundant 16S rRNA amplicon datasets have addressed the substantial technical variability introduced by sequencing protocols and, primarily, by the specific hypervariable region targeted. This variability often obscures true biological patterns and causes samples to cluster primarily by study rather than microbial similarity (67). Moreover, to combine datasets covering different hypervariable regions, it is necessary to perform some type of scaffolding. A common approach has been closed-reference OTU/ASV clustering, where sequences are matched to a reference database at a fixed similarity threshold, and unmatched reads are discarded. Another option is to collapse OTU/ASVs to a common taxonomic description to make them comparable across datasets. However, these methods have limitations: they cannot detect novel diversity beyond the reference set, focusing only on known taxa. In poorly characterised habitats, a substantial fraction of reads may be discarded, making the analysis heavily dependent on the completeness and resolution of the reference database (68). To overcome these limitations, phylogenetic placement approaches have been proposed as more accurate alternatives for integrating amplicon data across studies (**Table 1**).

One such tool is MaLiAmPi (57), which denoises sequences into ASVs, matches them to a full-length 16S rRNA allele repository and builds a *de novo* phylogenetic tree. Finally, it places taxa in the tree, and bins them into phylotypes based on phylogenetic similarity thresholds. An example of MaLiAmPi’s application is the Vaginal Microbiome Atlas during Pregnancy (69), an interactive R Shiny app that visualises 3,880 vaginal microbiome samples across 11 technically heterogeneous studies.

Another tool, SEPP (SATé-enabled Phylogenetic Placement) algorithm, available in a qiime2 plugin, handles inserting short sequence fragments directly into a broad, well-curated reference phylogeny, enabling meta-analysis of studies covering different hypervariable regions (58). SEPP was employed as an integrative step to enable diversity calculations across 15,096 diverse samples from the American Gut Project (70), facilitating direct comparison with previously generated datasets.

The development of these tools opens new perspectives for facilitating the analysis of community-generated data; however, although SEPP has not yet been applied to reproductive data integration, building specialised reference phylogenies for the reproductive tract is essential to advance its potential in the field.

## 5 Synthetic data generation

The generation of synthetic data is becoming a rapidly advancing field within the ML/DL domain and offers a powerful strategy to expand the capabilities of research in omics analyses. It is generally used to augment or balance available datasets and improve the capabilities of ML/DL models (e.g., phenotype classification). This approach is transformative because it allows researchers to create new high-fidelity data, statistically faithful and essentially indistinguishable from the original dataset.

Synthetic data, moreover, allows the exploration of rare or underrepresented patterns, helps preserve privacy when sharing data and enables more extensive evaluation and comparison of novel analytical tools (71). In conditions where sample size is limited and/or clinical conditions are rare, a common situation in clinical studies, augmenting datasets with synthetic data allows ML/DL to extract informative patterns that were previously obscured by the low number of samples.

Within the reproductive health landscape, where data acquisition is frequently problematic, synthetic data generation—particularly through statistical approaches—provides a critical use case for enabling much better training and robustness of predictive models. However, it is important to note that these advanced synthetic generation techniques have so far seen only limited application in reproductive microbiome research, and further investigation is needed to fully assess their potential in this field. Besides, the use of synthetic samples can lead to unrealistic benchmarking if researchers fail to maintain strict data separation during model evaluation. Data leakage across train/test boundaries occurs when generative models are trained prior to data splitting, allowing information from the test set to influence the training distribution. To prevent this, synthetic generation must be strictly isolated to the training set alone.

**Figure 4:**
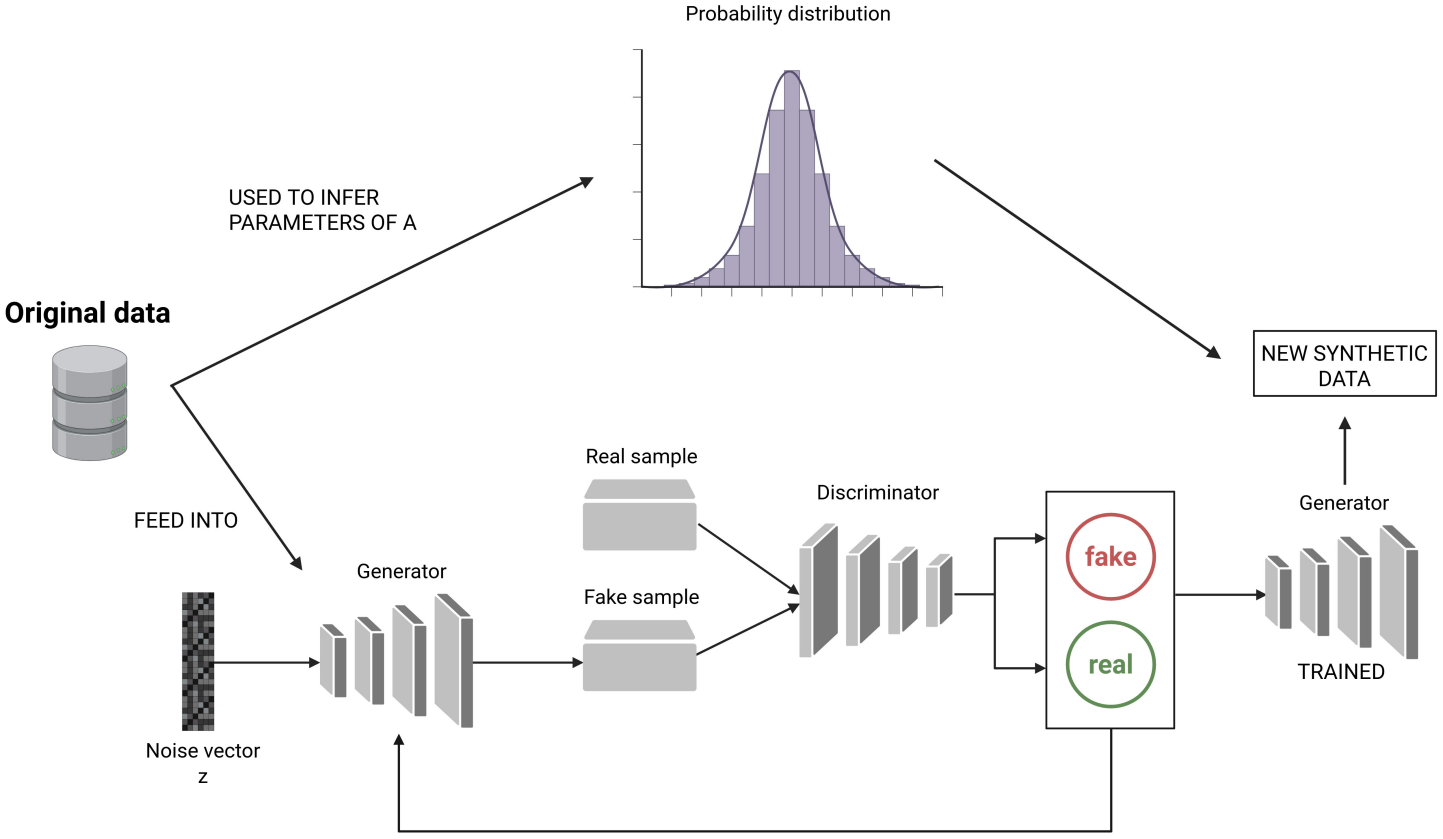
Two paradigms for synthetic data generation: one using original data to infer the parameters of a probability distribution and sample from it, and the other using a generative adversarial network, where the generator attempts to produce realistic data and the discriminator learns to distinguish between the real and generated data, resulting in a competitive training process. Created with Biorender.com.

Current methods for generating synthetic microbiome data (see **Figure 4**) can be classified into statistical model-based and DL generative approaches (**Table 2**), the former assuming a specific distribution and the latter learning it directly from the data. In both cases, the limits of generation fundamentally depend on the quality of the original data, with DL methods often requiring larger quantities of data to perform well.

**Table 2:** A collection of tools for generating synthetic microbiome data.

Category	Tool	Method	Availability
Statistical-based Models	metaSPARSIM [Patuzzi et al., (72)]	Species abundances via Gamma Distribution Technical variability via Hypergeometric Distribution	R
	MIDASim [He et al., (73)]	Presence-Absence Sparsity Modelling Relative abundance via Gaussian copula model	R
	TADA [Sayyari et al., (74)]	Incorporates phylogenetic information Models true and sampling variation via beta and binomial distributions	Python
DL Generative Models	MB-GAN [Rong et al., (75)]	Includes a phylogenetic transformation layer in the network. Earth Mover’s distance in the discriminator network	Python
	phylaGAN [Sharma et al., (76)]	Data generation via conditional GAN Learning latent representations using autoencoders Includes a custom phenotype classification pipeline	Python
DL Diffusion Model	MB-DDPM (Huo et al., (77))	Diffusion-Reverse Diffusion process U-Net-based architecture	Python

metaSPARSIM, Metagenomic Simulation of Sparse Microbial Communities; MIDASim, Microbiome Data Simulator; TADA, Tree-Aware Data Augmentation; MB-GAN, Microbiome Generative Adversarial Network; phylaGAN, Phylogenetic Generative Adversarial Network; MB-DDPM, Microbiome Denoising Diffusion Probabilistic Model.

Ready-to-use tools are available for statistical model-based approaches; for instance, metaSPARSIM specialises in generating realistic 16S rRNA count data, simulating it using gamma and hypergeometric distributions with parameters that can be estimated from the real data (72). Another tool, MIDASim (73), addresses this same augmentation challenge; it models data sparsity through a presence-absence step and then uses a Gaussian copula model to generate relative abundance data. Its utility has been demonstrated on pregnancy vaginal microbiome datasets, where the generated data successfully preserved the original samples’ alpha and beta-diversity measures (better than metaSPARSim and other methods), thus providing a larger, realistic dataset for downstream analysis of certain conditions and ML/DL classification models training (73). Moreover, by taking advantage of the biological structure of the data, we can improve the quality of synthetic data. TADA (74) incorporates biological knowledge by employing phylogenetic information to guide its hierarchical generative process. The benefit of this “smarter” generation was shown in comparisons to classic methods like ADASYN or SMOTE across two gut microbiome datasets, where ML models trained with TADA’s data were better at classifying patient phenotypes—detecting conditions like IBD or different body mass index categories directly from the microbiome data—compared to models trained using only the raw original data (74).

In contrast to statistical methods, DL methods such as Generative Adversarial Networks (GANs) learn data patterns in a competitive training process trying to generate data indistinguishable from the input (**Figure 4**). For example, MB-GAN (75) leverages GAN architectures incorporating phylogenetic information in a transformation layer of the network, producing gut microbiome simulations with similar sparsity, diversity, and taxa–taxa correlations to the original data. Furthermore, phylaGAN (76) is a tool that uses a conditional GAN and an autoencoder for data generation, providing a phenotype prediction pipeline based on a taxonomy-aware neural network approach. phylaGAN has been applied to metagenomic gut datasets from diabetes and cirrhosis patients, demonstrating improvements in classification performance: specifically, higher Area Under the Receiver Operating Characteristic Curve (AUC), specificity, and sensitivity compared to models trained on raw microbiome data (76).

A promising but underexplored approach in microbiome research is the use of diffusion models, which have emerged as state-of-the-art generative approaches across multiple domains due to their ability to model complex data distributions with high fidelity. Notably, MB-DDPM, a recent diffusion model, has been applied to the gut microbiome cohorts of obesity and IBD, accurately reproducing the bacterial community-level structure (77).

Although synthetic data can be a useful tool for data augmentation, dealing with data scarcity and imbalance and helping the training of ML/DL models, it comes with important limitations that must be carefully considered. Synthetic samples do not introduce new biological information and their quality is bounded by the quality, diversity, and representativeness of the original experimental data. They may fail to capture the full variability of real microbiome datasets, potentially introduce biases, and propagate errors if the original data quality is low and more benchmarking is needed to assess their usefulness, especially for phenotype classification tasks. Ultimately, the value of synthetic augmentation is limited without external validation cohorts to confirm its true biological and clinical applicability.

Additionally, in reproductive microbiome studies considering smaller cohorts, DL-based generative and diffusion tools are generally inappropriate, as training heavily parametrised models on small and sparse data increases the risk of overfitting rather than learning the biological distribution (71). While there is no strict numerical threshold, reflecting the cohort sizes successfully utilised in recent literature (75–77), datasets of approximately 300 to 500 samples are generally observed to support reliable model training and generalisable results. Therefore, the discussed statistical techniques remain more appropriate methods for smaller datasets.

## 6 Dimensionality reduction

Dimensionality reduction plays a central role in high-dimensional omic studies, particularly in microbiome. By reducing dimensionality, it becomes possible to filter redundant or irrelevant/noisy taxa and highlight the most biologically relevant ones that are truly related with the study condition. These approaches distill complex datasets into feature subsets that (i) capture the signal of interest, removing the background noise and (ii) allow finding potential biomarker candidates from the vast taxa landscape. In this context, two main strategies are commonly employed with different purposes: data transformation and biomarker signature identification (feature selection).

### 6.1 Data transformation

Data transformation techniques are used to project the data into a lower-dimensional space with new features called components or embeddings, while preserving the most informative aspects of the original data. This is particularly useful for data visualisation, as these techniques are able to project data into 2 or 3 coordinates, where groups of interest may be distinguishable visually if their samples’ microbial profiles cluster together. In fact, there is parallelism with beta-diversity analyses, as the usual techniques to visualise these similarities between samples are indeed Data Transformation methods (78). Further, these embeddings can also be used as input for ML/DL tasks; especially autoencoders’ latent space can serve as input for neural network classification pipelines (79).

Principal Component Analysis (PCA) may not be the most suitable approach, as it is based on the euclidean distance, which is not recommended for compositional data due to the spurious relationships between taxa. An alternative method is the robust Aitchison PCA (80), which employs a modified CLR normalisation (Aitchison distance) prior to PCA. This principle of using a compositional-aware metric before the actual transformation is the basis of their application to microbiome data (25).

Another method is PCoA, which projects the pairwise sample distances (e.g., Bray-Curtis, Unifrac) into a low-dimensional space, where the inter-sample relationships can be visualised. It generalises PCA, which is equivalent to performing PCoA using the Euclidean distance. PCoA has been actively applied in microbiome data analyses. For instance, it was applied using the Unifrac distance and separated the lower and higher reproductive tract community compositions of 544 samples across 110 women, highlighting their different microbial dynamics (81).

Unlike methods such as PCA and PCoA that primarily capture global variance or dissimilarities, non-linear techniques like UMAP and t-SNE (78, 82) excel at preserving local neighborhood structures, making them more effective for uncovering complex, non-linear patterns. UMAP is able to condense multiple sources of variability in fewer dimensions than PCoA, especially in the large datasets (78). It has been used in the Vaginal Microbiome Atlas during Pregnancy (69), interactively showing the distribution of samples across different phenotypes or metadata, displaying 3,880 vaginal microbiome samples from 1,402 pregnant individuals. Finally, a t-SNE model applied to 33 cervical microbiome profiles with UniFrac distance successfully separated a less severe endometriosis cluster and classified patients into three distinct groups according to their reproductive ability (83).

**Figure 5:**
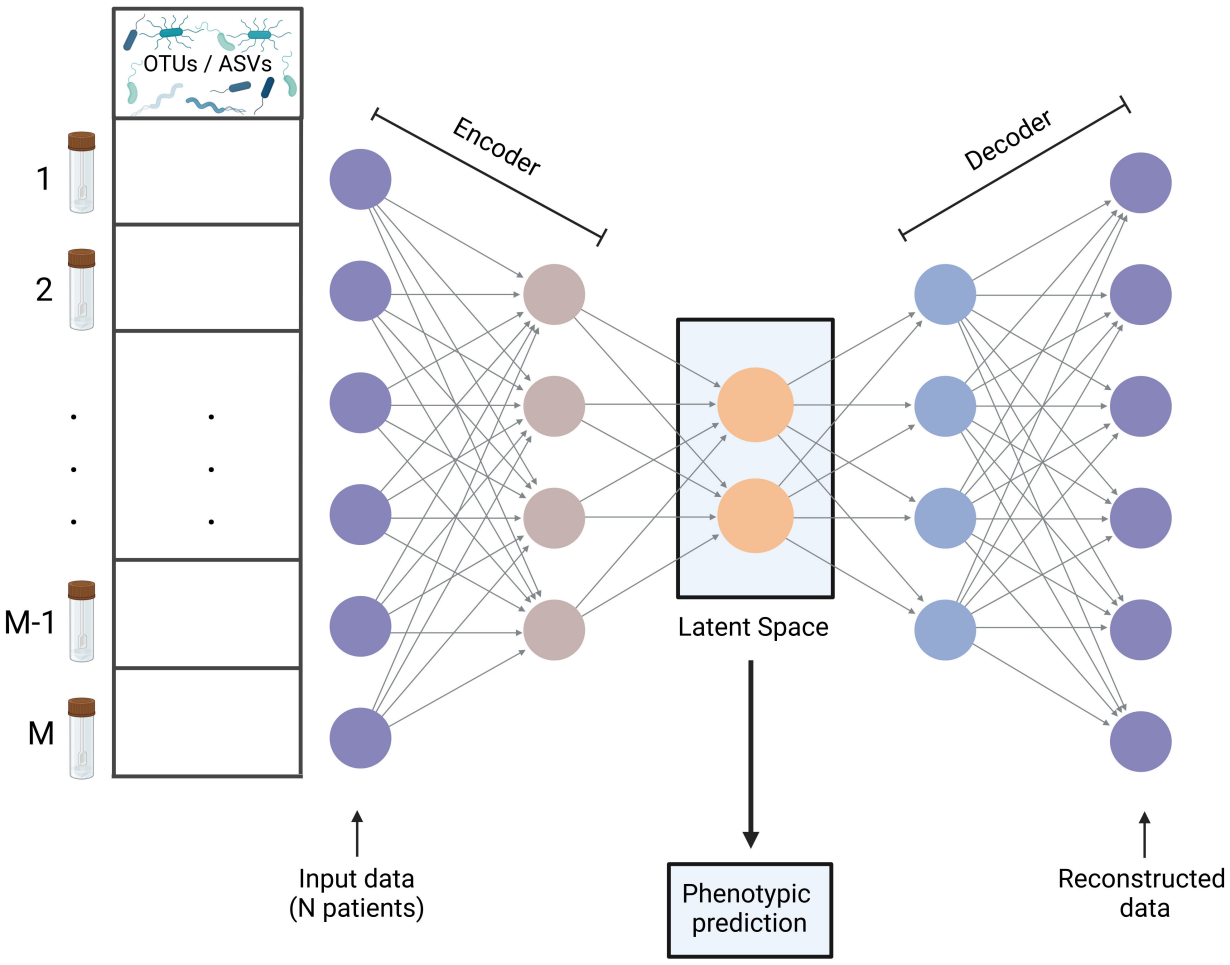
An autoencoder is a type of artificial neural network used for unsupervised learning. It consists of an encoder and a decoder, which are trained to reconstruct the input data. The encoder compresses the input data into a latent-space representation, while the decoder reconstructs the original input from this representation. This embedded representation can serve as input for downstream analyses. OTU (Operational Taxonomic Units); ASV (Amplicon Sequence Variants). Created with Biorender.com.

Among DL techniques, autoencoders stand out for their popularity in reducing the dimensionality of data to a lower-dimensional space called the latent space (**Figure 5**). A framework called DeepMicro that transforms the high-dimensional space into a robust low-dimensional representation using various types of different autoencoders has been proposed (79). Using these latent space representations, various classification algorithms were evaluated on publicly available human gut metagenomic datasets from six different disease cohorts: IBD, type 2 diabetes in European women, type 2 diabetes in Chinese individuals, obesity, liver cirrhosis, and colorectal cancer (79). The authors found that the DeepMicro representation outperformed traditional dimensionality reduction as a previous step before classification. This architecture is particularly promising for samples from low-biomass environments, such as those often found in the reproductive tract studies. In these contexts, autoencoders can learn general representations that effectively separate true biological signals from the high levels of background noise.

### 6.2 Biomarker signature identification/feature selection

Feature selection plays a crucial role in microbiome data analysis and ML/DL tasks, as datasets are composed of thousands of features, many of which may be irrelevant or redundant, potentially degrading model performance and interpretability. This step could be considered as a generalisation of differential abundance tests used to identify biomarkers before performing phenotype classification, where the discovered signatures should be evaluated in terms of predictive power. Unlike the previously mentioned Data Transformation techniques that map the original data to a new feature space, feature selection involves using the condition information to identify a small subset of essential features (e.g., biomarkers) by removing irrelevant or redundant information. ML feature selection techniques can be categorised into filter, wrapper and embedded approaches (84). Filter methods rank features based on their intrinsic properties, independently of the ML algorithm to be used afterward. Wrapper methods use the ML model classification performance to guide the selection, typically at a higher computational cost. Finally, embedded methods, with the well-known LASSO as an example (85), integrate feature selection directly into the classification model training process.

The amount of feature selection algorithms available in ML is vast, but their application to reproductive data remains limited. For instance, a very popular feature selection algorithm used in bioinformatics is mRMR (minimum Redundancy Maximum Relevance) (86). It is a filter method which has been extensively applied to find maximum relevance between the selected genes and the target variable as well as minimum redundancy among the identified genes. In microbiome, a previous study used mRMR with the gut microbiomes of individuals representing three distinct racial groups and was able to extract microbial genes that reflect differences among these races with a high accuracy of classification (87). Further, mRMR was used to identify 300 microbial biomarkers from a cohort of 806 Chinese individuals with various conditions (type 2 diabetes, rheumatoid arthritis, liver cirrhosis, and healthy controls), enabling a multiclass logistic regression model to achieve an average AUC of 0.9475—highlighting the power of this approach in discovering disease-related features from microbiome data (88).

Recent analyses on multiple gut disease-related microbiome datasets support these observations. In particular, feature selection paired with CLR normalisation consistently improved the performance of phenotype classification pipelines, especially when combined with Logistic Regression (28). Their study also revealed that mRMR and LASSO emerged as the most effective feature selection methods, outperforming alternatives in both predictive performance and compactness of the selected biomarker sets. This is particularly relevant in the context of vaginal microbiome data, where the dominance of *Lactobacillus* leads to highly skewed feature distributions. While such taxa may be consistently identified as highly relevant, mRMR’s minimum redundancy criterion promotes the selection of additional features that provide complementary, non-redundant information, enabling the model to capture a broader microbial landscape beyond the dominant signal.

The role of the endometrial microbiome in the context of recurrent implantation failure (RIF) and recurrent pregnancy loss (RPL) has received little attention, but it is now a subject of growing interest. Recent descriptive studies have demonstrated that women experiencing RIF or RPL often exhibit distinct dysbiotic endometrial profiles (89–91). This is a field that could benefit from feature selection techniques, as endometrial microbiome data is particularly susceptible to noise and contamination. Sequencing artifacts and low-abundance taxa can obscure biologically meaningful patterns. Feature selection helps to filter out the irrelevant or noisy features that can harm the performance of classification models. In this context, a recent investigation of RPL used SVM, LASSO and RF to select features by consensus, achieving to successfully isolate a core predictive microbial signature comprised of *Streptococcus, Chryseobacterium*, and *Fusobacterium* (90) that attained 0.762 AUC.

Although the male reproductive tract microbiome is less studied than the microbiome in female, recent studies are providing more proof of its role in male reproductive health. Similar to the endometrial environment, the seminal microbiome is characterised by low biomass (1), making feature selection highly useful for distinguishing true biological signatures from noise and background contamination. For example, a recent metagenomics study investigating seminal microbiome alterations utilised a Random Forest approach to successfully identify predictive microbial features. By ranking these features according to their contribution to classification accuracy, the researchers were able to identify an association between *Brevundimonas* and altered semen parameters (92).

Finally, genetic algorithms are wrapper methods that can be useful and high-performing for searching for the best combination of variables to build a prediction model, normally at a high computational cost. For example, a previous study applied a hybrid approach combining PCA and a genetic algorithm to identify informative gut bacterial taxa associated with obesity and metabolic syndrome (93). In their pipeline, the genetic algorithm used the disease information as guidance to select the most informative principal components for classification. Subsequently, the bacterial species contributing to these selected components were extracted as the most relevant taxa for distinguishing between conditions.

Altogether, feature selection methods demonstrate high potential to uncover meaningful microbial signatures and find signals that differential abundance analysis might miss, enhancing predictive modelling of health conditions.

## 7 Phenotype classification

Phenotype classification refers to the application of computational models to predict a host condition using microbial composition data as input. ML/DL algorithms automatically learn these patterns from training data and can then classify new, unseen samples based on the learned model. When applied to the intricate world of the microbiome, these models hold immense promise for inferring complex biological relationships, helping to identify how microorganisms interact with the host and in the early diagnosis of diseases. Nevertheless, their effectiveness relies on access to large, high-quality datasets, as their data-intensive nature requires substantial sample sizes to produce generalisable insights.

The adoption of classification techniques in microbiome is growing rapidly, as common bioinformatic programming languages provide accessible training and evaluation of classification models through powerful and constantly updated libraries such as caret (94) in R and scikit-learn (95) in Python, making these advanced analytical tools easy to handle for researchers.

**Figure 6:**
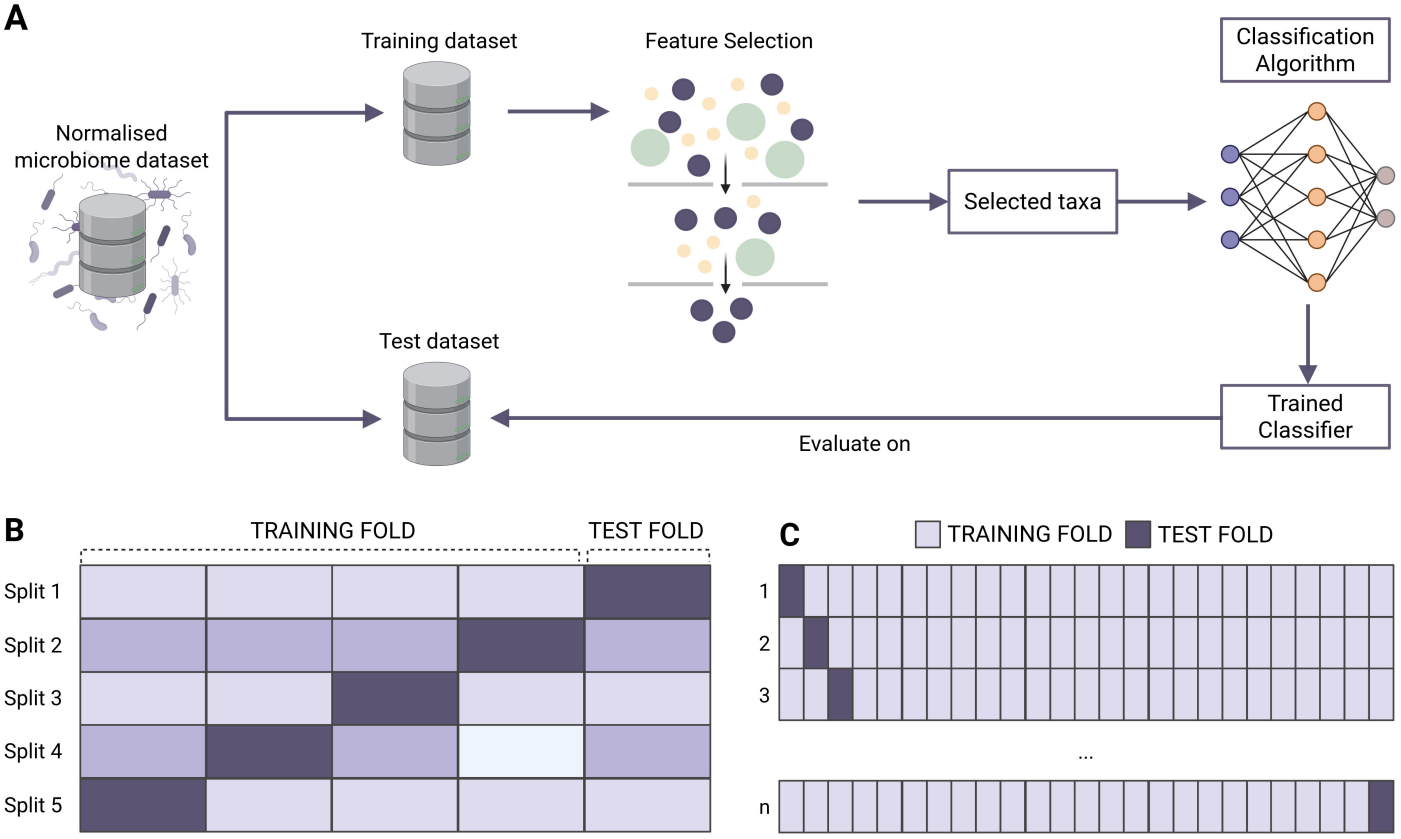
Validation of Feature Selection and Classifier Pipelines. **(A)** Normalised microbiome dataset used to generate training and test datasets. Feature selection is performed on the training dataset to identify relevant taxa, which are then used to train classification algorithms. The resulting trained classifier predictive performance is evaluated on the test dataset. **(B)** 5-Fold Cross-Validation strategy. **(C)** Leave-One-Out Cross-Validation strategy. Created with Biorender.com.

To rigorously evaluate the performance and generalisability of these classification models, techniques such as Hold-Out, Cross-Validation (CV) or Leave-One-Out (LOOCV) are essential (96). These methods represent a fundamental step in the ML/DL pipeline; they provide a robust framework to assess how the model will perform on unseen data. Moreover, this approach helps avoiding overfitting—a situation where the model memorises the training data rather than learning generalisable patterns. As shown in **Figure 6**, these strategies involve splitting the dataset into training and testing subsets one or multiple times, allowing models to be trained and validated under different data partitions to ensure obtaining an unbiased performance estimation.

However, these approaches must not only be accounted for by the classification step. A critical and frequent pitfall in microbiome predictive modelling is data leakage, which occurs when information from outside the training dataset influences the creation of the model, producing overly optimistic performance estimates. To avoid data leakage, any data-dependent processing step—such as differential abundance testing, feature selection, or the generation of synthetic data—must be strictly performed on the training set, independently within each fold of a CV procedure. In particular, another form of leakage can arise when multiple samples from the same subject are not properly treated; having the same subject in both the training and test splits leads to biased and inflated validation results. For longitudinal studies, this principle extends to maintaining strict chronological boundaries across the splits.

Furthermore, because microbiome datasets frequently suffer from highly skewed class distributions (e.g., controls heavily outnumbering diseased patients), ML pipelines must explicitly address class imbalance to ensure correct model training. This can be achieved through methods like weighted loss functions (penalising more misclassifications of the minority class), under-sampling, or over-sampling, again ensuring these techniques are performed solely to the training data (8). Beyond standard discrimination metrics like AUC, sensitivity and specificity should be used, model calibration is also crucial, as it ensures that the predicted probabilities accurately reflect the true likelihood of a phenotype and the certainty the model has on its prediction. For instance, a model that is only 60% confident in its prediction must be interpreted with caution and flagged for expert review. Additionally, identifying the specific conditions under which the model exhibits higher uncertainty provides valuable insights into its underlying logic, aiding in the interpretation of how the model works.

Finally, while CV and LOOCV provide rigorous internal validation, they do not guarantee real-world applicability. Microbiome data is particularly subject to batch effects, biases and population differences. Therefore, establishing the utility of a model requires external validation on independent cohorts. Once tested externally, the learned microbial signatures are proven to be generalisable, rather than overfitted to the characteristics and possible noise of a specific study cohort.

In microbiome research, a commonly used approach is LASSO regularised logistic regression, which combines classification with embedded feature selection. In general, this algorithm is often used to address multicollinearity and overfitting by adding a penalty term to the loss function. An example of its effectiveness is demonstrated in a previous study, where it was employed to identify taxa predictive of preterm births from the vaginal microbiome data (97). The algorithm was able to select bacterial species with predictive power that were excluded by traditional statistical tests.

Traditional algorithms such as Support Vector Machines (SVM) and Random Forest (RF) have also been applied in microbiome studies. For example, to explore the microbial signatures associated with endometriosis severity, Perrotta et al. (98) employed a RF classifier on 59 samples—a robust ensemble learning method that builds multiple decision trees using different subsets of features and samples, ultimately making predictions based on majority voting. The model achieved an AUC of 0.89, indicating high discriminatory power in distinguishing between early (rASRM stage 1–2) and advanced (rASRM stage 3–4) endometriosis. Notably, the relative abundance of a taxon *Anaerococcus* emerged as a key predictive feature during menstruation (98). Similarly, another study based on a smaller cohort of 11 women across 22 samples displayed the applicability of RF workflows to endometriosis detection using gut and vaginal samples. Although the authors acknowledged that this limited sample size increases the risk of overfitting, their findings were supported by an analysis of 88 publicly available samples, where the gut microbiome outperformed vaginal features predictive power (99).

Phenotype classifier benefits are also reflected in a previous study, where a RF classifier was developed using the relative abundances of vaginal microbiome and clinical metadata from 2017 women to predict vaginal infections, achieving an AUC of 0.83 (100). While overall model sensitivity was moderate, performance improved notably for BV, with 92% accuracy, showing that the characteristics of the vaginal microbiome of patients with BV were more distinguishable than those of other vaginal infections. These results denote RF ability to exploit the high-dimensional, yet informative microbiome features to classify BV patients.

Another relevant example is a study where vaginal microbiome composition and immune marker concentrations were assessed on 28 women (across 81 samples) to predict how they affect pregnancy outcomes in patients undergoing *in vitro* fertilisation (101). Using a SVM classifier, they achieved a F1-score of 0.9 using microbial features, close to perfect sensitivity and specificity. Moreover, an explainability analysis revealed that *Gardnerella vaginalis* was the most impactful variable, with high relative abundance negatively associated with pregnancy. Conversely, *Lactobacillus crispatus*, which ranked in the top ten features in two different models, was positively associated with pregnancy achievement (101). Applying this same SVM approach to other reproductive outcomes, recent work successfully predicted miscarriage in a cohort of 54 women (102). The models achieved strong classification performances, with an AUC of 0.85 using vaginal data. Although the small cohort size limits the generalisability of the results, the study provides another compelling example of how ML can be successfully applied to the reproductive microbiome.

Further, a large-scale meta-analysis integrating over 10,000 microbiome samples across gut, vaginal, and oral niches, developed binary classifiers to distinguish healthy individuals from those with gestational diabetes, polycystic ovary syndrome, and preterm birth (65). A diverse set of classical machine learning algorithms—including RF, Gradient Boosting, SVM, K-Nearest Neighbours, and LASSO—was employed, with models trained on 70% of the data and evaluated on a 30% held-out set. Ensemble learning was used to combine the predictions of the different models and improved predictive performance. Notably, the strongest results were observed for preterm birth (0.94 AUC), where predictive accuracy depended on gestational timing, with mid- and late-pregnancy data outperforming early-pregnancy samples. The incorporation of clinical and environmental metadata also enhanced model performance. Mothers experiencing preterm birth exhibited a notable increase in the alpha diversity of the vaginal microbiome, driven by a depletion of *Lactobacillus* species and an expansion of *Prevotella* (65).

A final technique worth mentioning to cover a wide landscape of models is TaxoNN (103). This framework organises OTUs into four clusters based on their phyla and applies an ensemble of NNs over them. It showed a consistent improvement in performance in simulations, cirrhosis, and diabetes datasets, compared to the next best-performing method that they had applied (103).

In summary, while ML/DL classifiers demonstrate preliminary success in identifying reproductive tract phenotypes—and although some reviews have highlighted the suitability of specific techniques over others across different microbiome body sites—the methodological foundation of current literature remains fragile. The pervasive reliance on scarce cohorts often yields overfitted models with inflated performance metrics driven by batch effects rather than biological signals. To transition from exploratory algorithms to actionable tools, the field must prioritise large-scale, harmonised, multi-centre datasets. Only coupling this robust data infrastructure with rigorous data-leakage prevention and external validation would enable to deploy generalisable models that can lead to research applications in reproductive care.

## 8 Explainable artificial intelligence

Although ML and DL models have advanced the field of microbiome research—particularly in studying the gut microbiome and its association with disease states and progression—their application faces a big limitation: the black-box nature of complex algorithms in the model’s decision-making process. While inherently interpretable models (e.g., logistic regression, decision trees) offer transparent decision rules, they typically sacrifice predictive power compared to more complex models such as RF or CNNs. Consequently, *post-hoc* XAI methods are essential to decipher how specific microbial species influence the algorithmic insights regarding an individual’s pathology (104).

When implementing XAI, it is critical to distinguish between global and local explanations, as they provide different information. Global explanations focus on the overall behaviour of the model, providing feature importance summaries that identify the broad microbial signatures associated with a condition. In contrast, local explanations elucidate the model’s decision-making process for an individual sample (105). For clinical applications, local, per-patient explanations are more valuable. They identify the specific microbial dysbiosis driving a particular patient’s diagnostic prediction, thereby potentially informing individual treatment decisions.

A prominent XAI framework used to generate both local explanations and global summaries is SHAP (SHapley Additive exPlanations) (106). However, applying SHAP to microbiome data requires careful methodological consideration. Standard SHAP approaches assume feature independence, an assumption that is violated by the spurious correlations arising from the compositional nature of microbiome data.

To address this, researchers must appropriately transform the data, applying the CLR transformation prior to model training and SHAP analysis to relax the compositional constraints. Furthermore, utilising specific implementations such as TreeSHAP—designed explicitly for tree-based models (e.g., RF, XGBoost)—can handle feature dependencies (107, 108). TreeSHAP computes exact Shapley values by following only valid decision paths within the trained trees, thereby reducing the risk of considering unrealistic feature combinations that are impossible under the microbiome compositional constraints. When these methodological caveats are respected, SHAP demonstrates improved reliability (109).

Similarly, in a recent study focused on cervical cancer, SHAP was employed in conjunction with a RF classifier to uncover discriminative microbial patterns in vaginal microbiome samples (109). SHAP identified that elevated levels of *Ralstonia* significantly contributed to the prediction of cervical cancer, while its lower abundance, along with that of *Pseudomonas, Streptococcus, and Brevundimonas*, correlated with healthy controls. To further enhance interpretability, the study utilised the DALEX (moDel Agnostic Language for Exploration and eXplanation) framework (110), available in both R and Python. DALEX helped visualise how specific microbial taxa influenced prediction probabilities.

To illustrate global explainability, feature importance metrics such as Mean Decrease in Impurity can provide summaries of which microbial taxa drive model predictions across a dataset. For example, a recent study utilising RF models investigated the link between the maternal vaginal microbiome and the early onset of Type 1 Diabetes in vaginally delivered children (111). By extracting global feature importance from their models, researchers identified specific bacterial and fungal groups characterising the dysbiosis associated with the disease development. This global explanation revealed that the fungal genus *Tylospora* possessed the highest predictive importance and was significantly more abundant in the vaginal mycobiome of mothers with T1D children. Additionally, bacterial features such as the genus *Aerococcus* and specific metabolic pathways related to amino acid biosynthesis emerged as globally important predictors (111).

XAI plays a vital role in the field of biomedical research currently, as it allows researchers to trust the predictions made by complex (often black box) models. These examples demonstrate that incorporating XAI into the design of microbiome pipelines facilitates the interpretation and validation of results, providing a powerful tool for clinical decision support. However, a more extensive evaluation of XAI approaches is still required to ensure clinically trustworthy explanations (112).

## 9 Discussion

Currently, microbiome studies in the reproductive tract primarily rely on descriptive statistical analyses, which have provided valuable understanding of its association with reproductive health. Nevertheless, studies investigating functional mechanisms beyond mere characterisation are lacking. Moving forward, the growing availability of carefully curated datasets will enable the deployment of predictive ML/DL models, which hold potential to assist clinicians in diagnosis and prognosis (97, 102). Promising advances are already being observed in areas such as preterm birth and endometriosis (81, 83, 97, 99). However, we must temper these expectations: whilst ML/DL techniques may reveal information that traditional statistical methods might overlook, true clinical utility will require thorough experimental validation to move from associative patterns to actionable causality and utility.

Throughout this review, we outline the computational methods currently available and the specific problems they can solve, rather than to perform benchmark comparisons, as these studies in the field of human reproduction are scarce and the necessary harmonised microbiome datasets are still limited. Moreover, we discuss specific reproductive microbiome challenges inherent to these data modalities and provide guidance to address them. A significant technical barrier is the inherently low microbial biomass nature of environments such as the upper female reproductive tract (e.g., uterus, fallopian tubes, or ovaries) and the male reproductive tract (e.g., seminal fluid or testicular tissue). In these niches, the biological signal is often sparse and can be easily eclipsed by background or (kit-derived) noise, which has direct implications for feature selection. Due to these constraints, 16S rRNA sequencing is the most suitable choice today for low-biomass sites, while still requiring the use of decontamination tools. As the lower female reproductive tract is dominated by the genus *Lactobacillus*, this predominance exacerbates issues of data compositionality and can create biased distributions in taxonomic tables, making both the CLR transformation and the use of a diverse set of evaluation metrics essential. Further, the physiological dynamics of the female reproductive system introduces structured temporal dependencies, such as hormonal fluctuations across menstrual cycles or pregnancy, which may act as confounding factors within the model. Similarly, in males, factors such as sexual abstinence duration can influence microbial load and seminal quality parameters, justifying their inclusion as relevant covariates. By leveraging XAI to disentangle these physiological confounders, researchers can more accurately isolate microbial signatures with true clinical potential. This becomes particularly relevant in studies integrating external or public datasets, where such metadata are often incomplete or unavailable, and controlling for these effects during study design is not always feasible.

It is also important to recognise that, while traditional ML algorithms, such as SVM and RF, have demonstrated preliminary maturity in classifying reproductive phenotypes (e.g., preterm birth), many of the advanced DL and multi-omic integration frameworks discussed here are heavily borrowed from the gut microbiome, oncology or chronic disease research. The transferability of these methods to reproductive data remains to be fully validated. For researchers entering this field, we advise a conservative approach: prioritise simpler, interpretable ML models combined with ensemble-based differential abundance and feature selection tools for small cohorts, reserving complex DL frameworks for large-scale, multi-centre meta-analyses once sufficient data becomes available.

To navigate the specific hurdles identified in this review, particularly the constraints imposed by characteristically small cohort sizes, we have synthesised an optimised analysis framework as detailed in **Figure 2**. This framework serves as a practical set of recommendations for dealing with such data limitations, helping to ensure that scientific rigour is maintained even when samples are scarce. We have further expanded upon this by establishing a direct link between key analytical objectives and their associated advantages, limitations, and evidence-based best practices (**Table 3**). By providing this structured overview alongside the available computational tools, we offer a solid foundation for future research and facilitate the effective application of machine and deep learning within the complex landscape of reproductive datasets.

**Table 3:** Strategic framework for the application of machine learning and deep learning in reproductive microbiome research, summarising key analytical goals, associated advantages, methodological limitations, recommended best practices, and representative computational tools.

Research goal	Tools	Advantages	Limitations	Recommendations
Biomarker Discovery (e.g., identifying taxa linked to infertility)	MRMR, Mutual Information, LASSO, ANCOM-BC, ALDEx2	Filters background noise, enhances predictive model performance and improves interpretability	Variability across tools.	Use ensemble methods to avoid tool bias Strictly confine feature selection to the training set to prevent data leakage
Patient Stratification/Advanced Exploratory Analysis (e.g., stratifying vaginal community types)	Aitchison PCA, UMAP, t-SNE, Autoencoders	Condenses complex, non-linear variability into 2D/3D visual embeddings	UMAP and t-SNE results can be sensitive to parameter choices.	Avoid Euclidean PCA due to compositionality Use Aitchison distances or non-linear methods to preserve complex local structures
Predictive Modelling (e.g. predict IVF outcome from microbial profiles)	Random Forest, SVM, LASSO DeepMicro, TaxoNN	Captures non-linear host-microbiome interactions for early diagnosis and personalised care.	Highly data-intensive; small cohort sizes carry the risk of overfitting and batch effect-driven biases.	Perform rigorous cross-validation and validate externally on independent cohorts to ensure generalisability. Use batch effect removal tools if integrating data from different sources. Consider synthetic data augmentation for small cohorts and class imbalance. Validate models externally on independent cohorts and use XAI tools.
Multi-omic Integration (Integrate multimodal data of the same subjects (e.g., microbiome, metabolome)	MOFA, HONMF, DIABLO, mmvec, JNBM, Autoencoders	Reveals cross-site and cross-kingdom interactions (e.g., microbe-metabolite, microbe-virus) and maximises the utility of small sample sizes	Sensitive to missing data and batch effects. Interpretability of latent factors can be challenging.	Ensure proper normalisation and scaling across omics layers. Use matched samples and address missing data before integration
Functional Analysis (e.g., Predict and quantify the metabolic potential of microbiomes)	PICRUSt2, HUMAnN, FMAP	Explains the mechanisms of disease. Functional traits can be used for explainable predictive modelling.	For 16S data, accuracy is fundamentally limited by the completeness of reference databases.	Transition from taxonomy to function. Use robust databases for 16S or direct shotgun metagenomics for higher resolution.

AUC-ROC, Area Under the Receiver Operating Characteristic curve; CLR, Centred Log-Ratio transformation; GAN, Generative Adversarial Network; HUMAnN, HMP Unified Metabolic Analysis Network; IVF, In vitro fertilisation; LASSO, Least Absolute Shrinkage and Selection Operator; LOOCV, Leave-One-Out Cross-Validation; ML/DL, Machine Learning/Deep Learning; NN, Neural Network; PCA, Principal Component Analysis; PCoA, Principal Coordinates Analysis; PICRUSt2, Phylogenetic Investigation of Communities by Reconstruction of Unobserved States; RF, Random Forest; SVM, Support Vector Machine; t-SNE, t-distributed Stochastic Neighbour Embedding; XAI, Explainable Artificial Intelligence.

Beyond methodological considerations, it is also important to contextualise the current limitations of ML/DL approaches in terms of clinical translation. The majority of the examples and studies reviewed currently support associative and predictive capabilities rather than verified mechanistic inference. True precision diagnostics and personalised reproductive care remain a long-term progressive goal. Current algorithms function as powerful tools that can highlight microbial dysbiosis or certain combinations of increase/depletion of taxa in a study group, but they do not immediately offer clinically actionable utility. The microbial signatures identified require extensive downstream experimental validation to prove biological causality.

A crucial element of this progression must be the integration of information at multiple levels. Primarily, harmonising heterogeneous microbiome datasets, ideally building curated data repositories that serve as powerful references to extract patterns from the highly variable microbial communities. In parallel, integrating multiple omics may uncover associations or intervention points that isolated microbiome data cannot provide. Alongside data integration, a key recommendation involves the incorporation of external biological knowledge (e.g., phylogenetic and functional information) into ML/DL models. This approach promises to enhance model performance, and significantly improve the interpretability of findings. Integrating this knowledge helps to mitigate the black-box nature of some models, making them easier to interpret and trust.

In conclusion, the integration of ML and DL into reproductive microbiome research represents a transformative step forward and a robust set of discovery tools. While true precision diagnostics remain a long-term goal, these computational methods are already laying the essential groundwork for future innovations in reproductive healthcare. Ultimately, by prioritising rigorous experimental validation and fostering multidisciplinary collaboration between clinicians and data scientists, the field can effectively bridge the gap between the *in silico* discoveries and potential clinical applications.

## Glossary

16S rRNA: 16S ribosomal Ribonucleic Acid
ANCOM-BC: Analysis of Compositions of Microbiomes with Bias Correction
ASVs: Amplicon Sequence Variants
AUC: Area Under the Curve
BV: Bacterial Vaginosis
CLR: Centered Log-Ratio
CNNs: Convolutional Neural Networks
ConQuR: Conditional Quantile Regression
DALEX: moDel Agnostic Language for Exploration and eXplanation
DEBIAS-M: Domain adaptation with phenotype Estimation and Batch Integration Across Studies of the Microbiome
DIABLO: Data Integration Analysis for Biomarker discovery using Latent variable Approaches for Omics studies
DL: Deep Learning
FDR: False Discovery Rate
GAN: Generative Adversarial Networks
HoCoRT: Host Contamination Removal Tool
HONMF: Hypergraph-induced Orthogonal Nonnegative Matrix Factorisation
HPV: Human Papillomavirus
IBD: Inflammatory Bowel Disease
ILR: Isometric Log Ratio
JNBM: Joint Negative Binomial Model
KEGG: Kyoto Encyclopedia of Genes and Genomes
LASSO: Least Absolute Shrinkage and Selection Operator
LOOCV: Leave-One-Out Cross-Validation
MAG: Metagenome-Assembled Genomes
MB-DDPM: Microbiome Denoising Diffusion Probabilistic Model
MB-GAN: Microbiome Generative Adversarial Network
metaSPARSIM: Metagenomic Simulation of Sparse Microbial Communities
MIDASim: Microbiome Data Simulator
ML: Machine Learning
MMUPHin: Meta-analysis Methods with Uniform Pipeline for Heterogeneity in Microbiome Studies
mmvec: Microbe Metabolite Vectors.
MOFA: Multi-Omics Factor Analysis
mRMR: Minimum Redundancy Maximum Relevance
NN: Neural Network
OTUs: Operational Taxonomic Units
PCA: Principal Component Analysis
PCoA: Principal Coordinate Analysis
phylaGAN: Phylogenetic Generative Adversarial Network
PLSDA-batch: Partial Least Squares Discriminant Analysis with batch correction
rASRM: Revised American Society of Reproductive Medicine
RF: Random Forest
RIF: recurrent implantation failure
RPL: recurrent pregnancy loss
SEPP: SATé-Enabled Phylogenetic Placement
SHAP: Shapley Additive Explanations
SVM: Support Vector Machine
TADA: Tree-Based Associative Data Augmentation
TaxoNN: Taxonomy-aware Neural Network
t-SNE: t-Distributed Stochastic Neighbor Embedding
UMAP: Uniform Manifold Approximation and Projection
VAE: Variational Autoencoder
VLR: Variance of the Log-Ratio
XAI: Explainable Artificial Intelligence.
