# Supplementary material for: Decoding the reproductive microbiome: enabling clinical and biological insights through machine and deep learning
Source: Front Endocrinol (Lausanne). 2026 Jun 15;17:1812407. doi: 10.3389/fendo.2026.1812407 (PMC13310787; doi:10.3389/fendo.2026.1812407)
Supplement: Supplementary file 1 [file Table1.docx]

**Supplementary Table 1.** Summary of microbiome studies included in the review, grouped by data integration, synthetic data generation, data transformation, feature selection, phenotype classification, and explainable AI (XAI). Columns report the article, algorithm/tool, objective or studied condition, sequencing approach, sampling site, number of samples, and whether the article treats specifically reproductive tract data.

| Section | Article | Algorithm / Tool | Objective / Studied Condition | Sequencing Approach | Sampling Site | Number of Samples | Field |
| --- | --- | --- | --- | --- | --- | --- | --- |
| Data Integration | Kim *et al.*, 2026  (52) | JNBM | Paired microbial interactions associated with menopausal status | 16S rRNA gene sequencing | Vaginal and Urine Microbiome | 68 women | Reproductive |
|  | Happel *et al.*, 2023  (61) | DIABLO | Human Papillomavirus Infection and Bacterial Vaginosis | 16S rRNA gene sequencing, metagenomics sequencing, cytokine measurements and viral metagenomics | Cervicovaginal Microbiome | 33 women | Reproductive |
|  | Bokulich *et al.*, 2022  (62) | mmvec | Disease State Prediction and Genital Inflammation pathologies | 16S rRNA gene sequencing, liquid chromatography-mass spectrometry and immunoproteome cytometric bead arrays | Vaginal Microbiome | 72 women | Reproductive |
|  | Xiao *et al.*, 2024  (65) | ConQuR | Predictive risk of Preterm Birth, Gestational Diabetes, and Polycystic Ovary Syndrome | 16S rRNA gene sequencing and Metagenomics | Vaginal, gut and oral Microbiomes | 10,378 samples from PTB patients, 570 GDM patients, 87 samples from PCOS patients | Reproductive |
|  | Austin *et al.*, 2025  (55) | DEBIAS-M | Multi-dataset diagnostic prediction of Cervical Intraepithelial Neoplasia | 16S rRNA gene sequencing and Metagenomics | Cervicovaginal Microbiome | 323 women (92 neoplasia and 231 controls) from 5 technically diverse studies | Reproductive |
|  | Parraga-Leo *et al.*, 2024  (69) | MaLiAmPi | Multi-dataset Atlas of the Vaginal Microbiome during Pregnancy | 16S rRNA gene sequencing | Vaginal Microbiome | 3,880 samples belonging to 1,402 pregnant women (2,779 term, 745 preterm and 356 early preterm deliveries) from 11 heterogeneous cohorts | Reproductive |
|  | Haak *et al.*, 2021  (59) | MOFA | Multi-omic analysis of microbiome disorders in antibiotic perturbation and sepsis patients | 16S and 18S rRNA gene sequencing and short-chain fatty acids spectroscopy | Gut Microbiome | 46 samples (33 ill patients and 13 controls) | Non-Reproductive |
|  | Ma *et al.*, 2023  (49) | HONMF | Patient clustering analysis of ICU patients, stable bronchiectasis and soil ecosystems | 16S rRNA gene sequencing, fungal ITS and VIDISCA-NGS | Gut Microbiome, sputum and soil | 33 / 166 / 48 | Non-Reproductive |
|  | Ma *et al.*, 2022  (54) | MMUPHin | Population structure discovery. Meta-analysis of Inflammatory Bowel Disease | 16S rRNA gene sequencing | Gut Microbiome | 5,094 samples across 10 technically diverse studies | Non-Reproductive |
|  | Yeo *et al.*, 2024  (66) | PLSDA-batch | Identification of consensus head and neck cancer-associated microbiota signatures | 16S rRNA gene sequencing | Tumours and Adjacent tissue Microbiome | 938 samples across 12 technically diverse studies | Non-Reproductive |
|  | McDonald *et al.*, 2018  (70) | SEPP | Insert short sequences (sOTUs) into a reference phylogenetic tree to enable diversity analyses in the American Gut repository. | 16S rRNA gene sequencing | Gut Microbiome | 15,096 samples from 11,336 participants | Non-Reproductive |
| Synthetic Data Generation | He *et al.*, 2024  (73) | MIDASim | Realistic synthetic microbiome data generation tested on pregnancy and Inflammatory Bowel Disease datasets | 16S rRNA gene sequencing | Vaginal and Gut Micriobiome | 517 / 146 | Reproductive |
|  | Patuzzi *et al.*, 2019  (72) | metaSPARSim | Simulating synthetic 16S count tables to be used for the assessment of tools for preprocessing and downstream analysis, such as count data normalization, zero-values imputation, differential abundance testing | 16S rRNA gene sequencing | Human and Animal Gut Microbiome | 40 / 110 | Non-Reproductive |
|  | Sayyari *et al.*, 2019  (74) | TADA | Statistical generative model with integrated phylogenetic information to create new samples augmenting existing ones. Tested in an Inflammatory Bowel Disease dataset. | 16S rRNA gene sequencing | Gut Microbiome | 1,359 samples | Non-Reproductive |
|  | Rong *et al.*, 2021  (75) | MB-GAN | Studying Generative Adversarial Network models for synthetic data generation. Tested in an Inflammatory Bowel Disease dataset | Metagenomics Sequencing | Gut Microbiome | 396 samples | Non-Reproductive |
|  | Sharma *et al*., 2024  (76) | PhylaGAN | Taxonomic-aware approach with a Generative Adversarial Network designed to extract features from OTU structured data. Tested in diabetes, cirrhosis and obesity studies. | Metagenomics Sequencing | Gut Microbiome | 344 / 332 / 253 | Non-Reproductive |
|  | Huo *et al.*, 2026  (77) | MB-DDPM | Diffusion model approach designed to generate microbiome data. Tested in Inflammatory Bowel Disease and Obesity cohorts. | 16S rRNA gene sequencing and Metagenomics Sequencing | Gut Microbiome | 396 / 281 | Non-Reproductive |
| Data Transformation | Chen *et al.*, 2017  (81) | UniFrac distance-based PCoA | Characterization of the microbiota continuum in the female reproductive tract. Relationship with uterine diseases (hysteromyoma, adenomyosis, infertility due to endometriosis) and the menstrual cycle. | 16S rRNA gene sequencing and species-specific real-time qPCR | Vagina (lower vagina, posterior fornix), cervical canal, endometrium, fallopian tubes, and peritoneal fluid microbiome. | 544 biological samples processed from 110 women of reproductive age (95 in the initial cohort and 15 validation subjects) | Reproductive |
|  | Chang *et al.*, 2022  (83) | t-SNE | Early diagnosis of pelvic endometriosis and assessment of reproductive capacity via dimensionality reduction | 16S rRNA gene sequencing | Cervical Microbiome | 33 profiles (23 patients with endometriosis and 10 healthy controls) | Reproductive |
|  | Zhang *et al.*, 2025  (90) | Feature selection by Ensemble: Random Forest feature importances, SVM Recursive Feature Elimination and LASSO | Biomarker discovery and causal identification in Recurrent Pregnancy Loss | 16S rRNA gene sequencing | Endometrial Microbiome | 122 women (89 in the recurrent pregnancy loss group and 33 controls) | Reproductive |
|  | Veneruso *et al.*, 2023  (92) | Random Forest feature importances | Identification of specific microbial signatures associated with poor semen quality and reproductive outcomes. | 16S rRNA gene sequencing | Seminal Fluid Microbiome | 20 men | Reproductive |
|  | Oh *et al.*, 2020  (79) | DeepMicro | Learning low-dimensional representations using autoencoders. Tested in Type 2 Diabetes, Inflammatory Bowel Disease, Colorrectal Cancer, Cirrhosis and Obesity cohorts. | Metagenomics Sequencing | Gut Microbiome | 110 / 96 / 344 / 253 / 232 / 121 | Non-Reproductive |
|  | Chen *et al.*, 2016  (87) | mRMR Feature Selection | Studying microbial genes able to assess diferences between races | Metagenomics Sequencing | Gut Microbiome | 1,267 women | Non-Reproductive |
|  | Wu *et al.*, 2018  (88) | mRMR Feature Selection | Microbial biomarker identification using feature selection methods in a cohort comprising individuals with metabolic, autoimmune, hepatic, and healthy conditions | Metagenomics Sequencing | Gut Microbiome | 806 samples (170 diabetes, 130 cirrhosis, 123 arthritis and 383 controls) | Non-Reproductive |
|  | Zhang *et al.*, 2019  (93) | Genetic algorithm and principal component analysis | Identifying sets of bacterial species associated with obesity and metabolic syndrome | 16S rRNA gene sequencing | Gut Microbiome | 127 patients (22 obese and 105 healthy-weight) | Non-Reproductive |
| Phenotype Classification | Park *et al.*, 2022  (97) | Multivariate Logistic Regression with LASSO selection | Predictive imminent or pathological risk for early Spontaneous Preterm Birth | 16S rRNA gene sequencing | Cervicovaginal Microbiome | 150 women (54 preterm births and 96 term births) | Reproductive |
|  | Perrotta *et al*., 2020  (98) | Random Forest Classification | Discrimination between early-stage (rASRM I–II) and advanced (rASRM III–IV) endometriosis using gut and vaginal microbiomes | 16S rRNA gene sequencing | Vaginal and faecal Microbiome | 59 women (35 endometriosis cases and 24 controls) | Reproductive |
|  | Zhao *et al.*, 2025  (99) | Random Forest Classification | To characterise vaginal and gut microbiome profiles in endometriosis patients and evaluate their diagnostic potential | Metagenomics Sequencing | Vaginal and rectal Microbiome | 22 samples from 11 women (5 women with endometriosis and 6 controls) + 88 public external cohort | Reproductive |
|  | Dong *et al.*, 2024  (100) | Random Forest Classification | To investigate infection-associated microbial signatures in a meta-analysis seting. To evaluate their potential for diagnosing vaginal dysbiosis | 16S rRNA gene sequencing | Vaginal Microbiome | 2,017 women including controls and women with 6 tipes of infections. | Reproductive |
|  | Gudnadottir *et al.*, 2026  (102) | Support Vector Machine | To develop a prediction model for miscarriage risk by integrating maternal microbiome data (vaginal/faecal) with clinical and reproductive history | Metagenomics Sequencing | Vaginal and faecal Microbiome | 268 women (105 controls, 34 miscarriage, 27 preterm birth and intrauterine fetal death, 102 history of recurrent pregnancy loss) | Reproductive |
|  | Sharma *et al*., 2020  (103) | TaxoNN | Studying a taxonomy-aware neural network approach to improve predictive accuracy. Tested in type 2 Diabetes and Cirrhosis datasets | Metagenomics Sequencing | Gut Microbiome | 344 / 332 | Non-Reproductive |
| Explainable Artificial Intelligence | Bar *et al.*, 2025  (101) | SHAP values | To assess associations between vaginal microbiota, inflammatory markers and pregnancy outcomes | 16S rRNA gene sequencing and cytokines/chemokines | Vaginal Microbiome | 81 samples from 28 women (4 with unexplained infertility and 14 with male factor infertility) | Reproductive |
|  | Sekaran *et al.*, 2023  (109) | SHAP and DALEX visualiser | To analyse vaginal microbial composition across cervical cancer stages and identify signatures associated with disease pathogenesis | 16S rRNA gene sequencing | Vaginal Microbiome | 119 women (65 cervical cancer patients and 54 controls) | Reproductive |
|  | Ruotsalainen *et al.*, 2022  (111) | Random Forest global feature importances using Mean Decrease in Impurity | To validate maternal vaginal microbiome and mycobiome associations with gestational diabetes and analyse predicted metabolic pathway signatures. | 16S rRNA gene sequencing and ITS2 rDNA sequencing | Vaginal Microbiome | 49 women (25 women with at least one child with T1D by vaginal delivery and 24 control mothers with at least one vaginal delivery and no diagnosed child/children with diabetes) | Reproductive |

Note: ConQuR (Conditional Quantile Regression), DALEX (moDel Agnostic Language for Exploration and eXplanation), DEBIAS-M (Debiasing Microbial Association inference), DIABLO (Data Integration Analysis for Biomarker discovery using Latent Components for Omics studies), HONMF (Hypergraph induced Orthogonal Nonnegative Matrix Factorization model), JNBM (Joint Negative Binomial Model), LASSO (Least Absolute Shrinkage and Selection Operator), MaLiAmPi (Maximum Likelihood Amplicon Pipeline), MB-DDPM (Microbiome Denoising Diffusion Probabilistic Model), MB-GAN (Microbiome Generative Adversarial Network), metaSPARSIM (Metagenomic Simulation of Sparse Microbial Communities), MIDASim (Microbiome Data Simulator), MMUPHin (Meta-analysis Methods with Uniform Pipeline for Heterogeneity in Microbiome Studies), mmvec (Microbe–Metabolite Vectors), MOFA (Multi-Omics Factor Analysis), mRMR (Minimum Redundancy Maximum Relevance), OTUs (Operational Taxonomic Units), PCA (Principal Component Analysis), PCoA (Principal Coordinate Analysis), phylaGAN (Phylogenetic Generative Adversarial Network), PLSDA-batch (Partial Least Squares Discriminant Analysis with batch correction), rASRM (Revised American Society of Reproductive Medicine), SEPP (SATé-Enabled Phylogenetic Placement), SHAP (Shapley Additive Explanations), SVM (Support Vector Machine), TADA (Tree-Based Associative Data Augmentation), TaxoNN (Taxonomy-aware Neural Network), t-SNE (t-Distributed Stochastic Neighbor Embedding).
